# In vitro cytotoxic effect of stigmasterol derivatives against breast cancer cells

**DOI:** 10.1186/s12906-023-04137-y

**Published:** 2023-09-11

**Authors:** Nondumiso Premilla Dube, Vuyelwa Jacqueline Tembu, Getrude R Nyemba, Candace Davison, Goitsemodimo Herckious Rakodi, Douglas Kemboi, Jo-Anne de la Mare, Xavier Siwe-Noundou, Amanda-Lee Ezra Manicum

**Affiliations:** 1 Department of Chemistry, Tshwane University of Technology, Private Bag X680, Pretoria, 0001 South Africa; 2 Department of Biochemistry and Microbiology, Female Cancers Research at Rhodes University (FemCR2U), Makhanda/Grahamstown, 6140 South Africa; 3 Department of Physical Sciences, University of Kabianga, Kericho, 2030 Kenya; 4 Department of Pharmaceutical Sciences, Sefako Makgatho Health Sciences University, Pretoria, 0204 South Africa

**Keywords:** Stigmasterol, Cytotoxicity, MCF-7, HCC70, MCF-12A

## Abstract

**Background:**

Stigmasterol is an unsaturated phytosterol that belong to the class of tetracyclic steroids abundant in *Rhoicissus tridentata*. Stigmasterol is an important constituent since it has shown impressive pharmacological effects such as anti-osteoarthritis, anticancer, anti-diabetic, anti-inflammatory, antiparasitic, immunomodulatory, antifungal, antioxidant, antibacterial, and neuroprotective activities. Furthermore, due to the presence of *π* system and hydroxyl group, stigmasterol is readily derivatized through substitution and addition reactions, allowing for the synthesis of a wide variety of stigmasterol derivatives.

**Methods:**

Stigmasterol (**1**) isolated from *Rhoicissus tridentata* was used as starting material to yield eight bio-active derivatives (**2**–**9**) through acetylation, epoxidation, epoxide ring opening, oxidation, and dihydroxylation reactions. The structures of all the compounds were established using spectroscopic techniques, NMR, IR, MS, and melting points. The synthesized stigmasterol derivatives were screened for cytotoxicity against the hormone receptor-positive breast cancer (MCF-7), triple-negative breast cancer (HCC70), and non-tumorigenic mammary epithelial (MCF-12 A) cell lines using the resazurin assay.

**Results:**

Eight stigmasterol derivatives were successfully synthesized namely; Stigmasterol acetate (**2**), Stigmasta-5,22-dien-3,7-dione (**3**), 5,6-Epoxystigmast-22-en-3*β*-ol (**4**), 5,6-Epoxystigmasta-3*β*,22,23-triol (**5**), Stigmastane-3*β*,5,6,22,23-pentol (**6**), Stigmasta-5-en-3,7-dion-22,23-diol (**7**), Stigmasta-3,7-dion-5,6,22,23-ol (**8**) and Stigmast-5-ene-3*β*,22,23-triol (**9**). This is the first report of Stigmasta-5-en-3,7-dion-22,23-diol (**7**) and Stigmasta-3,7-dion-5,6,22,23-ol (**8**). The synthesized stigmasterol analogues showed improved cytotoxic activity overall compared to the stigmasterol (**1**), which was not toxic to the three cell lines tested (EC_50_ ˃ 250 µM). In particular, 5,6-Epoxystigmast-22-en-3*β*-ol (**4**) and stigmast-5-ene-3*β*,22,23-triol (**9**) displayed improved cytotoxicity and selectivity against MCF-7 breast cancer cells (EC_50_ values of 21.92 and 22.94 µM, respectively), while stigmastane-3*β*,5,6,22,23-pentol (**6**) showed improved cytotoxic activity against the HCC70 cell line (EC_50_: 16.82 µM).

**Conclusion:**

Natural products from *Rhoicissus tridentata* and their derivatives exhibit a wide range of pharmacological activities, including anticancer activity. The results obtained from this study indicate that molecular modification of stigmasterol functional groups can generate structural analogues with improved anticancer activity. Stigmasterol derivatives have potential as candidates for novel anticancer drugs.

**Supplementary Information:**

The online version contains supplementary material available at 10.1186/s12906-023-04137-y.

## Introduction

*Rhoicissus tridentata* is a deciduous shrub in the Vitaceae family, widely distributed throughout the Eastern region of southern Africa [1]. It is widely used in African medicinal systems to treat various ailments including erectile dysfunction, pains, swelling, cuts, wounds, kidney and bladder complications, stomach ailments, and livestock diseases, as well as for gynaecological purposes [2–7]. The medicinal uses of *R. tridentata* were attributed to the presence of bioactive compounds such as terpenoids, alkaloids and flavonoids, which showed promising anti-cancer, antioxidant, and anti-inflammatory activities [7–12]. The aqueous root extract of *R. tridentata* had the strongest antiproliferative effect, inhibiting HepG2 cell growth by 96.27%, while the methanol extract inhibited proliferation by 87.01% [2]. Modification of natural product structures leads to the discovery of novel agents with improved cytotoxicity in resistant tumours, decreased toxicity, and improved solubility [13, 14]. In this study, the roots of *R. tridentata* were phytochemically investigated and afforded stigmasterol in good yield. Stigmasterol was then selected as a candidate for synthetic modification with the aim of enhancing the anticancer activity.

**Fig. 1:**
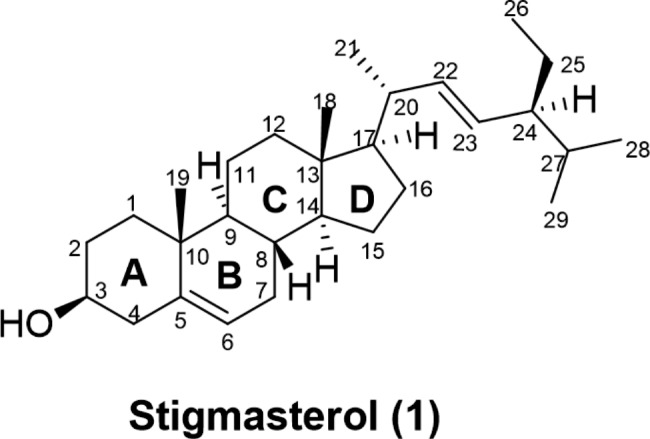
Chemical structure of Stigmasterol (**1**)

Stigmasterol is an unsaturated phytosterol that belongs to the steroids class. It is a secondary metabolite that was isolated for the first time in 1906 in Calabarbohne by Adolf Wind Form and A. Hauth [15], and has since been isolated as the common compound from various medicinal plants [16]. Stigmasterol (stigmasta-5,22-dien-3*β*-ol, C_29_H_48_O), also known as Wulzen anti-stiffness factor or stigmasterin, is characterized by the presence of a hydroxyl group in position C-3 of the steroid skeleton. It also has double bonds in positions 5, 6 of the B ring, and in position 22, 23 in the alkyl substituent, as well as an isoprenyl tail (Fig. 1**)** [15, 16].

Stigmasterol is used in various chemical manufacturing processes which are designed to generate numerous synthetic and semi-synthetic compounds for the pharmaceutical industry [15, 17]. It serves as a precursor in the synthesis of hormones such as progesterone as well as an intermediate in the biosynthesis of androgens, corticoids, oestrogens [18] and in the synthesis of vitamin D_3_ [15].

Stigmasterol has been investigated for its pharmacological prospects such as anti-osteoarthritic, immunomodulatory, cytotoxicity, antitumor, antiparasitic, antibacterial, antifungal, anti-diabetic, antimutagenic, antioxidant, anti-inflammatory and neuroprotective effects via in vitro and in vivo assays and molecular docking studies [15, 16, 19–23]. These studies unveiled the potential of stigmasterol as a key drug lead compound in the management of various illnesses, particularly in cancer therapy. In recent years, the anticancer potential of stigmasterol on numerous cancers was revealed through the different potential mechanisms of action [16]. This compound suppresses the development of various cancers, including gastric cancer [24], hepatoma [25], endometrial adenocarcinoma [26], breast cancer [26, 27], cholangiocarcinoma [28], skin cancer [29], gall bladder carcinoma [30], cervical cancer [27] and prostate cancer [31].

Stigmasterol isolated from the *Typhonium flagelliforme* mutant plant was found to be more effective against the human breast cancer MCF-7 cell line with an IC_50_ value of 0.1623 µM than cisplatin with an IC_50_ value of 13.2 µM [32, 33]. Stigmasterol can induce oxidative stress in MCF-7 cells, which leads to apoptosis. A competitive activator with a single high-affinity binding site on FXR and hydrophobic interactions facilitates this [32]. Li et al. [24] reported that stigmasterol suppressed proliferation and induced apoptosis in the human gastric cancer cell line SNU-1 through modulation of the JAK/STAT signalling pathway with an IC_50_ value of 15 µM. According to Bae et al. [34], stigmasterol can reduce the growth of human ovarian cancer cells ES2 and OV90 by 50% at a treatment concentration of 20 µg/mL, dose-dependently inducing apoptosis of ovarian cancer cells by causing mitochondrial malfunction, ROS production, and calcium overload in the mitochondria and cytosol.

In addition to stigmasterol, some of its derivatives, including stigmasta-5,22-dien-3,7-dione (**3**), 5,6-epoxystigmasta-3*β*,22,23-triol (**5**) and stigmast-5-ene-3*β*,22,23-triol (**9**) were also tested for their pharmacological aspects and showed promising activity. Stigmasta-5,22-dien-3,7-dione (**3**) was screened for its immunomodulatory potential by Donkwe et al. [35]. Compound **3** displayed moderate inhibitory activity on the oxidative burst of the whole blood (IC_50_ = 15.6 ± 2.1 µM) compared to the control ibuprofen (IC_50_ = 12.1 µM). In a study conducted by O’Callaghan et al. [36] to evaluate the cytotoxicity of oxidized derivatives of stigmasterol in the U937 human monocytic cell line using the Fluorescein Diacetate/Ethidium Bromide (FDA/EtBr) staining assay, 5,6-epoxystigmasta-3*β*,22,23-triol (**5**) and stigmast-5-ene-3*β*,22,23-triol (**9**), were found to induce a significant (p < 0.05) level of cell death as compared to the untreated control. Stigmast-5-ene-3*β*,22,23-triol (**9**) caused a significant reduction (p < 0.05) in cell viability at each of the three concentrations employed (30, 60, and 120 µM), reducing cell viability to 52.3% at the highest concentration. The 5,6-epoxystigmasta-3*β*,22,23-triol (**5**) significantly reduced cell viability (p < 0.05) at the 60 and 120 µM concentrations. The mode of cell death following staining with Hoechst 33,342 was identified as apoptosis in cells incubated with 5,6-epoxystigmasta-3*β*,22,23-triol (**5**) [36].

Furthermore, because of the pharmacophores present, such as alkene bonds and the hydroxyl group, Stigmasterol is readily derivatized through substitution and addition reactions, allowing for the synthesis of a wide variety of stigmasterol derivatives. As a result, stigmasterol was selected as a candidate for synthetic modification. Eight derivatives of Stigmasterol (**1**) were successfully synthesized via acetylation, oxidation, epoxidation and ring-opening and dihydroxylation of the epoxide, to enhance their anticancer activity. The generated products were characterized using spectroscopic techniques and screened for anticancer activity against the MCF-7 and HCC70 breast cancer cell lines in comparison to the MCF-12 A non-cancerous breast epithelial cell line.

## Methods and materials

### General experimental procedures

Column chromatography was performed using silica gel (Kieselgel 40–63 μm, Macherey-Nagel, Germany). Thin Layer Chromatography (TLC) was carried out on Kieselgel-60 F 254 (Merck) 20 × 20 cm aluminium sheets for monitoring the compounds. 1D and 2D NMR data were recorded on a Varian Gemini 400 spectrometer at 400 MHz and 100 MHz for ^1^H and ^13^C nuclei, respectively at room temperature. All compounds were dissolved in deuterated chloroform (CDCl_3_). Chemical shifts for signals were reported in parts per million (ppm) on the delta scale (δ), referenced to tetramethyl silane as the internal standard. The chemical shifts were referenced at δ_H_ 7.26 ppm in ^1^H and δ_C_ 77.04 ppm in ^13^C NMR for CDCl_3_. Spin-spin coupling constants (*J*) were calculated in Hertz (Hz) and the splitting patterns were recorded as follows: *s* = singlet; *d* = doublet; *t* = triplet; *q* = quartet; *dd* = doublet of doublets; *m* = multiplet and *b* = broad.

Melting points (MP) were determined using a digital melting point apparatus (Stuart® SMP 20, Cole-Parmer, Staffordshire, UK), operated at 50 Hz and 75 W power and optical rotation was recorded on a Jasco P-2000 Polarimeter (JASCO, Germany). Infrared spectroscopy (IR) was carried out on a Fourier transform Spectrophotometer (PerkinElmer UATR Two, USA), in the range 400 cm^− 1^ to 4000 cm^− 1^ with a resolution of 4 cm^− 1^ and 32 scans. The high-resolution electron spray ionization mass spectroscopic (HR-ESI-MS) data were acquired on a Bruker Daltonics Compact QTOF mass spectrometer in positive mode (ESI^+^) using an electrospray ionization probe. The mass spectrometer was coupled to a thermal scientific ultimate 3000 Dionex UHPLC system consisting of an RS Auto Sampler WPS-3000, Pump HPG-3400 RS and detector DAD-3000 RS, using an Acclaim RSLC 120 C18, 2.2 μm, 2.1 × 100 mm (P/N 068982) column at 40 ºC, flow rate 0.2 ml/min, solvent: Water-Acetonitrile (10:90, v/v) each solvent containing 0.1% of formic acid, isocratic condition, 5 min run. Processed spectra of all reported compounds are shown in supplementary materials 1.

### Plant material

The plant *Rhoicissus tridentata* subsp. *cuneifolia* was purchased from a wildflower nursery in Hartbeespoort, S 05°04.579′ E 044°35.033′, North West province of South Africa. The plant specimen was identified by Erich Van Wyk and a voucher specimen was deposited at the South African National Biodiversity Institute (SANBI) in Pretoria (number 22,033).

### Extraction and isolation

Dried and grounded roots plant material (1864.36 g) of *R. tridentata* was extracted sequentially with *n*-hexane, dichloromethane (DCM), ethyl acetate (EtOAc) and methanol (MeOH) for 48 h at temperatures between 15 and 25 °C, using a shaker. The extracts were filtered and concentrated under reduced pressure at 35 °C on a rotary evaporator to obtain a yield (2.59 g *n*-hexane, 3.61 g DCM, 4.55 g EtOAc and 71.28 g MeOH). Based on TLC analysis, the DCM crude extract was then subjected to repeated column chromatography (CC) using silica gel mixed with *n*-hexane and eluted with solvent systems, *n*-hexane: DCM (9:1, 8:2, 7:3 and 5:5 v/v). A total of 200 fractions were collected into 100 ml beakers and concentrated to dryness under a fume hood. Fraction F13D from the DCM crude extract showed interesting compounds with R_f_ values of 0.110 and 0. 231 as major compounds. This fraction afforded colorless crystals of sitosterol and 73.4 mg white powder of stigmasterol (**1**).

### In vitro**cytotoxicity assay**

#### The resazurin assay

The cell viability after treatment with the Stigmasterol derivatives was assessed using the resazurin assay [37]. The triple negative breast cancer cell line (TNBC), HCC70 cells [oestrogen receptor (ER)-, progesterone receptor (PR)-, human epidermal growth factor-2 (HER-2)-] (ATCC: CRL-2315), was maintained in culture in RPMI-1640 media supplemented with 10% (v/v) heat-inactivated foetal bovine serum (FBS), 100 mg/ml streptomycin, 100 U/ml penicillin, 12.5 mg/ml amphotericin (PSA), 2 mM GlutaMAXTM and 2,5% (v/v) sodium bicarbonate. The hormone receptor-positive (ER^+^, PR ^+^, HER-2^−^), MCF-7 breast cancer cell line (ATCC: HTB-22) was maintained in Dulbecco’s Modified Eagle’s Medium (DMEM) supplemented with 10% (v/v) FBS, 100 U/ml PSA and 2 mM GlutaMAX™. The non-tumorigenic breast epithelial cell line MCF-12 A (ATCC: CRL-10,782) was maintained in DMEM:Hams F12 (1:1 ratio) supplemented in 10% (v/v) FBS, 500 ng/ml hydrocortisone, 100 U/ml PSA, 100 ng/ml cholera toxin, 10 mg/ml insulin and 20 ng/ml human epidermal growth factor (hEGF). All cells were maintained at 37 °C in a humidified 9% CO_2_ incubator. The HCC70, MCF-7 and MCF-12 A cells were seeded at 5000 cells/well in a 96 well plate and were left overnight in a 9% CO_2_ incubator at 37 °C to adhere. The cells were then treated with the synthetic compounds at a concentration range from 0.16 to 500.00 µM or with a vehicle control [0.2% (v/v) DMSO] for 96 h at 37˚C in a 9% CO_2_ incubator. Thereafter, into each well, a solution of 10 µL of a 0.54 mM resazurin was added, followed by incubation for 2–4 h. Solubilization solution (10% (w/v) SDS in 0.01 M HCl) was then added overnight. 0.54 nM of resazurin solution was added and the cells were incubated for 2–4 h at 37˚C in a 9% CO2 incubator. The fluorescence was then measured on a Spectramax spectrophotometer with excitation and emission wavelength set at 560 and 590 nm respectively. The experiment was repeated in technical triplicate and the data was analysed using GraphPad Prism Inc, (USA) with half-maximal effective concentration (EC_50_ values) determined by non-linear regression. Selectivity index values for the compounds were calculated as follows: (EC_50_ of compound against MCF12A cells) ÷ (EC_50_ of compound against breast cancer cells) where a SI *>* 1 is indicative of selective toxicity towards cancer cells vs. non-cancerous cells.

### Methods for the synthesis of stigmasterol derivatives

#### Acetylation procedure of stigmasterol

Pyridine (0.14 mL) and acetic anhydride (6 mL) were added to compound **1** (Stigmasterol) (0.739 g, 1.7907 mmol) in a 50 mL round-bottomed flask. The mixture was left to stir for 24 h at room temperature. The reaction was quenched with deionized water (10 mL) and was extracted with DCM (3 × 10 mL). The organic phase was washed with 2 M HCl (3 × 10 mL) to wash away excess pyridine and dried over anhydrous magnesium sulphate, filtered, and concentrated in *vacuo*. The residue was purified by column chromatography using *n*-Hexane: ethyl acetate (EtOAc) (98:2%) to afford compound **2** [38, 39].

#### Oxidation procedure of stigmasterol

To a solution of compound **1** (1.002 g, 2.4280 mmol) in acetone (24.28 mL) at 0 °C, Jones reagent (CrO_3_/H_2_SO_4_) (1.94 mL) was added drop-wise. The reaction mixture was stirred at temperatures between 15 and 25 °C for 22 h. Isopropyl alcohol was added dropwise to remove excess Jones reagent, indicated by the appearance of a deep green colour. Upon completion, 5 mL of water was added before extraction with diethyl ether (2 × 30 mL). The combined ether layers were washed twice with sodium bicarbonate and brine, dried over anhydrous magnesium sulphate, and concentrated *in vacuo* [40]. The residue was purified by column chromatography using *n*-Hex: EtOAc (80:20%) to give compound **3** [39].

#### Epoxidation of stigmasterol

To a stirred solution of compound **1** (2.003 g, 4.8535 mmol) in DCM (49 mL) at temperatures between 15 and 25 °C was added *meta*-chloroperbenzoic acid (*m*CPBA) (4.1879 g, 24.2676 mmol). The reaction mixture was stirred for 24 h under N_2_ inert conditions. 5 mL of water was then added to the reaction mixture and extracted with DCM (2 × 30 mL) [39]. The organic phase was washed successively with 10% aqueous sodium metabisulphite (3 × 10 mL) and saturated sodium bicarbonate (3 × 20 mL), dried over anhydrous sodium sulphate, and concentrated *in vacuo*. The crude product was purified by column chromatography using *n*-Hex: EtOAc (95:5%) to give compound **4**.

#### Dihydroxylation of compound 4 (5,6-epoxystigmast-22-en-3β-ol)

To a mixture of *N*-methylmorpholine-*N*-oxide.2H_2_O (0.56 g, 4.7540 mmol), deionized water (2.9 mL), acetone (1.1 mL) and osmium tetroxide (21 µL) was added to the epoxide **4** (1.019 g, 2.3770 mmol). The reaction was initially slightly exothermic and was maintained at temperatures between 15 and 25 °C with a water bath. After stirring overnight at room temperature under nitrogen, the reaction was complete. 5 mL of sodium sulphite was used to quench the reaction. The reaction mixture was then extracted with EtOAc (2 × 30 mL). The organic phase was washed successively with saturated sodium bicarbonate (3 × 20 mL) and brine (30 mL), dried over anhydrous sodium sulphate, and concentrated *in vacuo*. The crude product was purified by column chromatography using *n*-Hex: EtOAc (80:20%) to give compound **5** [41].

#### Epoxide (4) ring-opening

To a suspension of the epoxide (compound **4**) (0.5860 g, 1.3670 mmol) in THF (9.1 mL) and H_2_O (5.5 mL) at temperatures between 15 and 25 °C, HClO_4_ (70% in H_2_O, 120 µL) was added. After 50 min, NaHCO_3_ (20 mL, saturated) was added, and the aqueous layer was extracted with ether (4 × 20 mL). The combined organic extracts were washed with saturated sodium bicarbonate (30 mL) and brine (30 mL), dried over anhydrous sodium sulphate and evaporated to give a white oil. The crude product was purified by column chromatography using *n*-Hex: EtOAc (80:20%) to give compound **6** [42].

#### Oxidation of compound 6 (stigmastane-3β,5,6,22,23-pentol)

To a solution of compound **6** (0.453 g, 0.9430 mmol) in acetone (9.4 mL) at 0 °C was added Jones reagent (CrO_3_/H_2_SO_4_) (0.38 mL) in a drop-wise manner. The reaction mixture was stirred at temperatures between 15 and 25 °C for 22 h. Isopropyl alcohol was added dropwise to remove excess Jones reagent, indicated by the appearance of a deep green colour. Upon completion, 5 mL of water was added before extraction with ether (2 × 30 mL). The combined ether layer was washed twice with sodium bicarbonate and brine, dried over anhydrous magnesium sulphate, and concentrated *in vacuo* [40]. The residue was purified by column chromatography using *n*-Hex: EtOAc (80:20%) to give compound **7**, compound **8** and compound **9**.

### Spectral data

#### Stigmasterol (1)

White powder, mp; 165–168 °C, [*α*]_D_^25^ +64.34 (CHCl_3_, Conc = 4.51 (w/v %), [M + H]^+^*m/z* 413.2688 (C_29_H_48_O) calculated for *m/z* 412.3705. IR; 2929 cm^− 1^, 2858 cm^− 1^ (C-H), 1464 cm^− 1^ (C = C), 3426 cm^− 1^ (OH). ^1^H NMR (CDCl_3_, 400 MHz), δ_H_ (*ppm*); 3.51 (*m*, H-3), 5.04 (*dd, J =* 8.50 Hz, 15.0 Hz, H-23), 5.17 (*dd, J =* 8.60 Hz, 15.8 Hz, H-22), 5.34 (*d, J =* 5.40 Hz, H-6), 0.69 (*s*, 3 H-18), 0.83 (*d*, *J* = 6.6 Hz, 3 H-27), 0.81 (*d*, *J* = 7.3 Hz, 3 H-26), 1.07 (*dd*, *J* = 13.4 Hz, 4.8 Hz, 3 H-21) and 0.80 (*t*, *J* = 7.2 Hz, 3 H-29). ^13^C-NMR (CDCl_3_, 100.6 MHz), δc (*ppm*); 37.2 (C-1), 31.6 (C-2), 71.8 (C-3), 42.3 (C-4), 140.7 (C-5), 121.7 (C-6), 31.9 (C-7), 31.6 (C-8), 50.1 (C-9), 36.5 (C-10), 21.1 (C-11), 40.5 (C-12), 42.2 (C-13), 56.8 (C-14), 24.3 (C-15), 29.3 (C-16), 56.1 (C-17), 12.2 (C-18), 19.7 (C-19), 40.5 (C-20), 21.2 (C-21), 138.3 (C-22), 129.2 (C-23), 51.2 (C-24), 33.9 (C-25), 19.3 (C-26), 21.1 (C-27), 25.4 (C-28), 12.0 (C-29).

#### Stigmasterol acetate (2)

^1^H NMR (400 MHz, CDCl_3_, δ *ppm*): 4.52 (1H, m, 3-H), 5.31 (1H, d, *J =* 5.1 Hz, 6-H), 5.06 (1H, dd, *J* = 15.2, 8.6 Hz, 22-H), 4.98 (1H, dd, *J =* 15.1, 8.5 Hz, 23-H), 1.96 (3H, s, 2’-H).

^13^C NMR (100.6 MHz, CDCl_3_, δ *ppm*): 37.01 (C-1), 28.92 (C-2), 74.00 (C-3), 42.22 (C-4), 139.66 (C-5), 122.65 (C-6), 31.90 (C-7), 31.87 (C-8), 50.06 (C-9), 36.61 (C-10), 21.23 (C-11), 38.13 (C-12), 40.51 (C-13), 56.79 (C-14), 21.45 (C-15), 25.42 (C-16), 55.94 (C-17), 12.06 (C-18), 19.32 (C-19), 39.64 (C-20), 21.10 (C-21), 138.33 (C-22), 129.29 (C-23), 51.25 (C-24), 27.78 (C-25), 21.02 (C-26), 19.00 (C-27), 24.37 (C-28), 12.26 (C-29), 170.56 (C-1’), 21.02 (C-2’).

#### Stigmasta-5,22-dien-3,7-dione (3)

^1^H NMR (400 MHz, CDCl_3_, δ *ppm*): 6.16 (1 H, s, 6-H), 5.11 (1 H, dd, *J* = 15.2, 8.5 Hz, 22-H), 5.04 (1 H, dd, *J =* 15.2, 8.5 Hz, 23-H).

^13^C NMR (100.6 MHz, CDCl_3_, δ *ppm*): 39.01 (C-1), 34.17 (C-2), 199.44 (C-3), 46.76 (C-4), 161.03 (C-5), 125.43 (C-6), 202.26 (C-7), 46.76 (C-8), 50.98 (C-9), 39.80 (C-10), 24.01 (C-11), 35.52 (C-12), 42.39 (C-13), 55.74 (C-14), 25.36 (C-15), 28.66 (C-16), 56.64 (C-17), 12.06 (C-18), 17.49 (C-19), 40.35 (C-20), 21.15 (C-21), 137.77 (C-22), 129.75 (C-23), 51.22 (C-24), 31.83 (C-25), 20.85 (C-26), 18.96 (C-27), 24.01 (C-28), 12.22 (C-29).

#### 5,6-Epoxystigmast-22-en-3β-ol (4)

^1^H NMR (400 MHz, CDCl_3_, δ *ppm*): 3.84 (1 H, tt, *J* = 10.8, 4.8 Hz, 3-H), 2.83 (1 H, d, *J* = 4.3 Hz, 6-H), 5.06 (1 H, dd, *J* = 15.2, 8.5 Hz, 22-H), 4.93 (1 H, dd, *J =* 15.2, 8.5 Hz, 23-H).

^13^C NMR (100.6 MHz, CDCl_3_, δ *ppm*): 34.88 (C-1), 29.90 (C-2), 68.73 (C-3), 42.61 (C-4), 65.72 (C-5), 59.30 (C-6), 32.42 (C-7), 31.10 (C-8), 51.22 (C-9), 39.32 (C-10), 21.10 (C-11), 39.89 (C-12), 42.24 (C-13), 56.97 (C-14), 24.13 (C-15), 28.82 (C-16), 55.64 (C-17), 12.06 (C-18), 18.99 (C-19), 40.47 (C-20), 20.65 (C-21), 138.23 (C-22), 129.34 (C-23), 51.22 (C-24), 31.88 (C-25), 21.18 (C-26), 15.94 (C-27), 28.76 (C-28), 12.25 (C-29) [39].

#### 5,6-Epoxystigmasta-3β,22,23-triol (5)

^1^H NMR (400 MHz, CDCl_3_, δ *ppm*): 3.88 (1 H, tt, *J* = 11.2, 4.8 Hz, 3-H), 2.89 (1 H, d, *J* = 4.4 Hz, 6-H), 3.68 (1 H, dd, m, 22-H), 3.48 (1 H, m, 23-H).

^13^C NMR (100.6 MHz, CDCl_3_, δ *ppm*): 38.67 (C-1), 29.89 (C-2), 65.71 (C-3), 42.67 (C-4), 63.04 (C-5), 62.06 (C-6), 32.39 (C-7), 31.04 (C-8), 56.45 (C-9), 34.85 (C-10), 20.82 (C-11), 39.81 (C-12), 48.25 (C-13), 59.25 (C-14), 20.92 (C-15), 29.12 (C-16), 58.55 (C-17), 11.83(C-18), 19.35 (C-19), 39.19 (C-20), 15.89 (C-21), 69.33 (C-22), 68.61 (C-23), 55.76 (C-24), 29.12 (C-25), 15.89 (C-26), 16.24 (C-27), 20.92 (C-28), 12.34 (C-29).

#### Stigmastane-3β,5,6,22,23-pentol (6)

^1^H NMR (400 MHz, CDCl_3_, δ *ppm*): 4.09 (1 H, dd, *J* = 11.5, 6.3 Hz, 3-H), 3.53 (1 H, s, 6-H), 2.73 (1 H, dd, *J* = 7.2, 2.3 Hz, 22-H), 2.49 (1 H, m, 23-H).

^13^C NMR (100.6 MHz, CDCl_3_, δ *ppm*): 30.27 (C-1), 38.26 (C-2), 67.66 (C-3), 38.56 (C-4), 76.07 (C-5), 75.85 (C-6), 20.15 (C-7), 43.03 (C-8), 53.51 (C-9), 55.53 (C-10), 20.90 (C-11), 43.08 (C-12), 55.56 (C-13), 56.13 (C-14), 29.11 (C-15), 29.32 (C-16), 45.68 (C-17), 12.13 (C-18), 12.42 (C-19), 48.28 (C-20), 16.06 (C-21), 63.10 (C-22), 62.12 (C-23), 48.76 (C-24), 19.35 (C-25), 29.32 (C-26), 19.35 (C-27), 30.27 (C-28), 16.83 (C-29).

#### Stigmasta-5-en-3,7-dion-22,23-diol (7)

^1^H NMR (400 MHz, CDCl_3_, δ *ppm*): 6.16 (1 H, s, 6-H), 2.75 (1 H, d, *J* = 2.4 Hz, 22-H), 2.74 (1 H, d, *J =* 2.3 Hz, 23-H).

^13^C NMR (100.6 MHz, CDCl_3_, δ *ppm*): 38.92 (C-1), 39.75 (C-2), 199.35 (C-3), 46.70 (C-4), 160.84 (C-5), 125.52 (C-6), 202.06 (C-7), 42.81 (C-8), 46.70 (C-9), 35.51 (C-10), 20.92 (C-11), 34.16 (C-12), 39.75 (C-13), 53.26 (C-14), 29.15 (C-15), 29.33 (C-16), 50.90 (C-17), 11.88 (C-18), 12.40 (C-19), 34.16 (C-20), 16.02 (C-21), 61.87 (C-22), 56.16 (C-23), 48.27 (C-24), 19.61 (C-25), 29.33 (C-26), 20.13 (C-27), 29.67 (C-28), 16.02 (C-29).

#### Stigmasta-3,7-dion-5,6,22,23-ol (8)

^1^H NMR (400 MHz, CDCl_3_, δ *ppm*): 3.16 (1 H, *m*, 6-H), 2.89 (1 H, s, 22-H), 2.85 (1 H, s, 23-H).

^13^C NMR (100.6 MHz, CDCl_3_, δ *ppm*): 37.40 (C-1), 41.80 (C-2), 211.07 (C-3), 43.13 (C-4), 82.67 (C-5), 82.65 (C-6), 211.33 (C-7), 43.34 (C-8), 55.84 (C-9), 37.40 (C-10), 20.91 (C-11), 38.44 (C-12), 43.34 (C-13), 55.86 (C-14), 29.09 (C-15), 20.93 (C-16), 62.00 (C-17), 11.94 (C-18), 12.38 (C-19), 37.42 (C-20), 13.80 (C-21), 62.92 (C-22), 62.03 (C-23), 58.59 (C-24), 19.61 (C-25), 29.09 (C-26), 20.12 (C-27), 29.09 (C-28), 15.98 (C-29).

#### Stigmast-5-ene-3β,22,23-triol (9)

^1^H NMR (400 MHz, CDCl_3_, δ *ppm*): 4.33 (1 H, t, *J* = 2.8 Hz, 3-H), 5.80 (1 H, s, 6-H), 2.75 (1 H, d, *J* = 2.3 Hz, 22-H), 2.73 (1 H, d, *J* = 2.3 Hz, 23-H).

^13^C NMR (100.6 MHz, CDCl_3_, δ *ppm*): 34.22 (C-1), 29.68 (C-2), 73.13 (C-3), 42.79 (C-4), 168.43 (C-5), 126.30 (C-6), 29.17 (C-7), 29.72 (C-8), 53.56 (C-9), 37.09 (C-10), 20.15 (C-11), 38.56 (C-12), 42.79 (C-13), 56.03 (C-14), 24.37 (C-15), 20.95 (C-16), 55.52 (C-17), 11.98 (C-18), 16.07 (C-19), 37.05 (C-20), 12.43 (C-21), 63.00 (C-22), 62.04 (C-23), 48.28 (C-24), 19.60 (C-25), 20.95 (C-26), 20.15 (C-27), 24.37 (C-28), 16.07 (C-29).

## Results and discussion

**Scheme 1:**
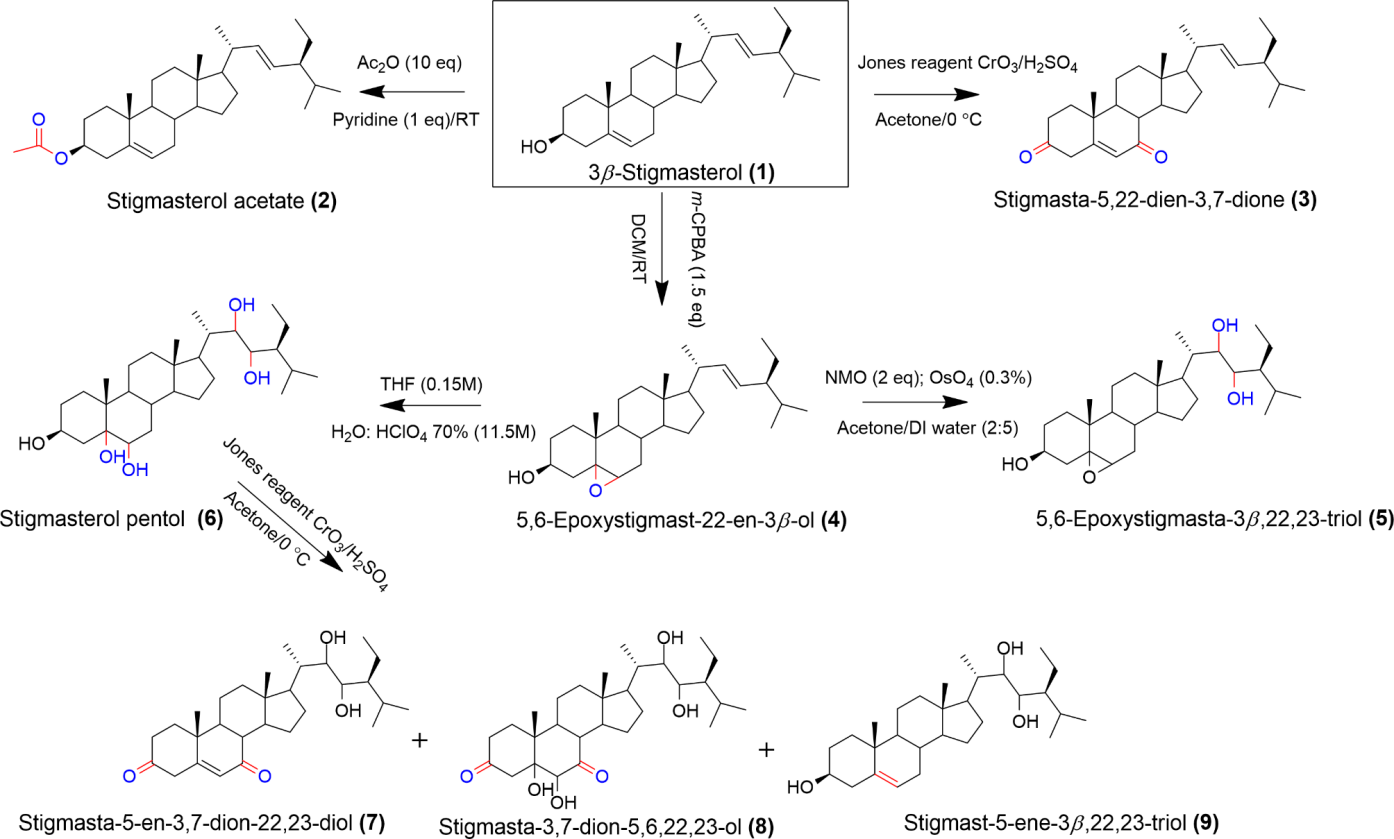
Schematic representation of the synthesized stigmasterol derivatives

Analogues of stigmasterol were synthesized with the aim of enhancing its anticancer activity through acetylation, epoxidation, epoxide ring opening, oxidation, and dihydroxylation reactions as shown in Scheme 1. The synthesis of the stigmasterol derivatives was possible because of the pharmacophores present in stigmasterol, such as alkene bonds in position C-5, C-6, and position C-22, C-23 in the alkyl substituent and the hydroxyl group in the position C-3.

### Characterization of synthesized compounds

**Fig. 2:**
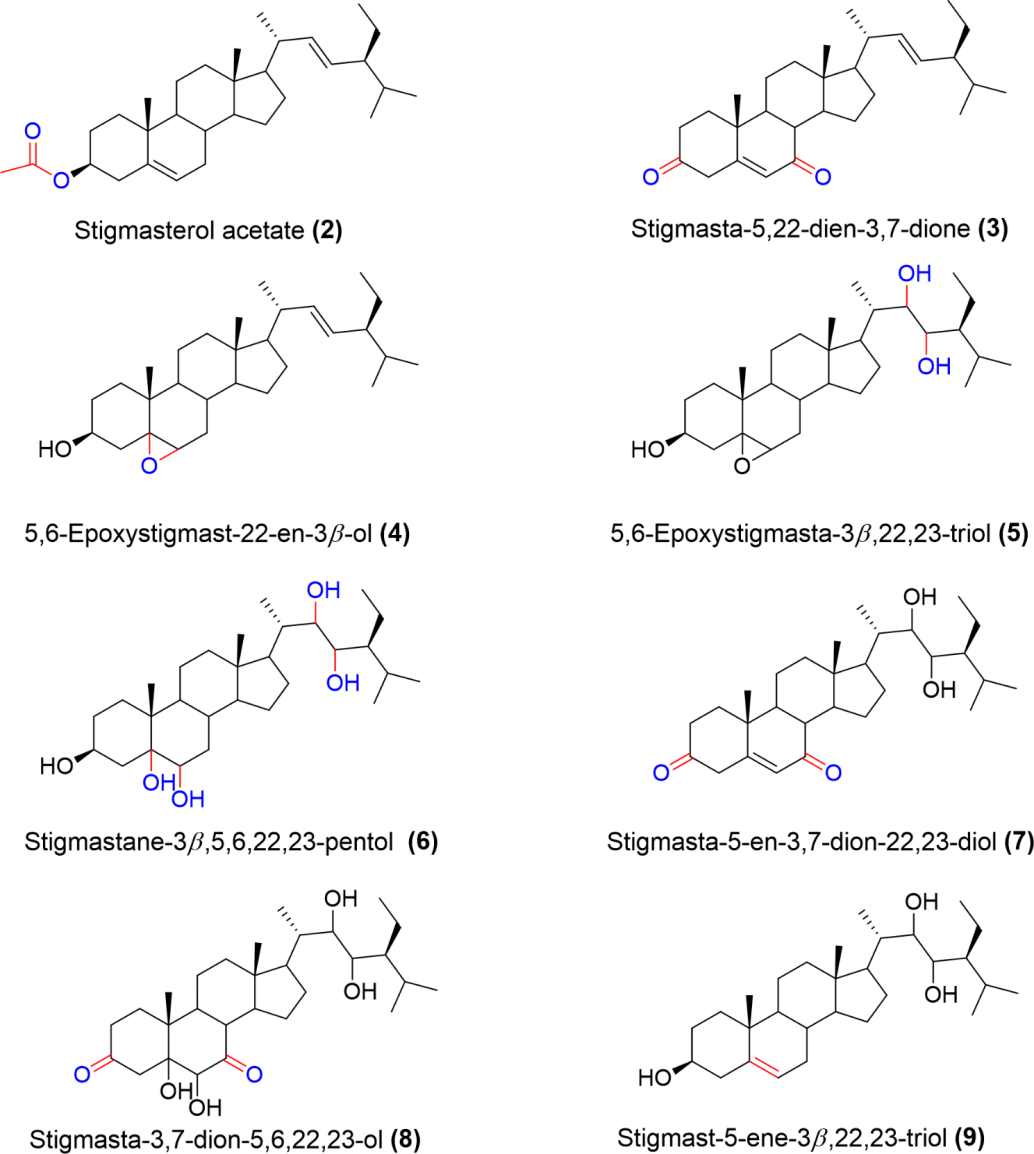
Structures of synthesized stigmasterol derivatives

Eight stigmasterol derivatives were successfully synthesized namely; Stigmasterol acetate (**2**), Stigmasta-5,22-dien-3,7-dione (**3**), 5,6-Epoxystigmast-22-en-3*β*-ol (**4**), 5,6-Epoxystigmasta-3β,22,23-triol (**5**), Stigmastane-3*β*,5,6,22,23-pentol (**6**), Stigmasta-5-en-3,7-dion-22,23-diol (**7**), Stigmasta-3,7-dion-5,6,22,23-ol (**8**) and Stigmast-5-ene-3*β*,22,23-triol (**9**). The structures of the synthesized compounds are shown in Fig. 2.

**Stigmasterol acetate** (**2**) was obtained as a white solid (0.5323 g, 1.1715 mmol, 65%) with a melting point of 139–141 °C. The specific optical rotation was recorded as [α]_D_^18.0^= -54.5° (c = 2.73 (w/v%), CHCl_3_). The Q-TOF mass spectrum showed a molecular ion [M-Na]^−^ peak at *m/*z 431.3788 with the molecular formula of C_31_H_50_O_2_. The IR spectrum showed a peak appearing at 2942.2 cm^− 1^ attributed to C-H stretches, the (C = O) stretch was observed at 1729.2 cm^− 1^, the (C = C) stretch was observed at 1461.0 cm^− 1^ and the absorption bands at 1026.2 cm^− 1^ were due to a (C-O) stretching.

Compound **2** was confirmed by the shift of the oxymethine proton (H-3) from the upfield region at δ_H_ 3.45 ppm (tt, *J* = 10.4, 4.3 Hz, 1 H) to a slightly downfield region at δ_H_ 4.52 ppm (m, 1 H) in the ^1^H NMR spectrum assigned to H-3, confirming an acetate group. In the ^13^C NMR spectrum a presence of downfield carbon resonance at δ_C_ 170.56 ppm was observed, typical of an acetate group. The NMR data of the derivative is comparable to the literature values reported by Foley et al. [39] for stigmasterol acetate (**2**).

**Stigmasta-5,22-dien-3,7-dione (3)** was obtained as a brown solid (0.4628 g, 1.1278 mmol, 46%) with a melting point of 152–155 °C. The specific optical rotation was recorded as [α]_D_^20.5^= -86.7° (c = 1.00, CHCl_3_). The Q-TOF mass spectrum showed a molecular ion [M + H]^+^ peak at *m/*z 425.3418 with the molecular formula of C_29_H_44_O_2_. The absorption band at 2942.2 cm^− 1^ in the IR spectrum was attributed to C-H stretches, the (C = O) stretch was observed at 1690.0 cm^− 1^ and the (C = C) stretch was observed at 1461.0 cm^− 1^.

The ^1^H NMR spectrum of the compound exhibited a sharp and long singlet at δ_H_ 6.16 ppm, attributed to the olefinic methine proton H-6. There was a chemical shift of the methine proton at position 6 from δ_H_ 5.28 ppm (d, *J* = 4.9 Hz, 1 H) to δ_H_ 6.16 ppm. The absence of the oxymethine proton at δ_H_ 3.45 ppm (tt, *J* = 10.4, 4.3 Hz, 1 H) of stigmasterol, in the ^1^H NMR spectrum confirmed the formation of a carbonyl at position C-3 displaying a carbon resonance at δ_C_ 199.44 ppm. Moreover, the difference in the ^13^C NMR spectra of the product and the starting material (stigmasterol) was the appearance of the signals at δ_C_ 199.44 and δ_C_ 202.26 ppm in the ^13^C NMR spectrum of the product. The peaks were attributed to the keto carbonyl carbons at C-3 and C-7, respectively [39]. The proton at δ_H_ 2.48 ppm (H-4) was seen correlating to C-3 which confirmed the assignment of the carbonyl to C-3. These values are consistent with those reported by Donkwe et al. [35].

**5,6-Epoxystigmast-22-en-3*β*-ol (4)** was obtained as a clear oil (1.5641 g, 3.6513 mmol, 75%) with a melting point of 142–145 °C. The specific optical rotation was recorded as [α]_D_^20.7^ = -60.2° (c = 1.25, CHCl_3_). The Q-TOF mass spectrum showed a molecular ion [M + H]^+^ peak at *m/*z 429.3719 with the molecular formula of C_29_H_48_O_2_. The IR spectrum showed a broad (-OH) peak appearing at 3452.3 cm^− 1^, the absorption band at 2935.7 cm^− 1^ was attributed to C-H stretches and the (C = C) stretch was observed at 1729.2 cm^− 1^.

The disappearance of the olefinic proton at δ_H_ 5.28 ppm (d, *J* = 4.9 Hz, 1 H) in H-6 of stigmasterol in the ^1^H NMR spectrum and the appearance of the carbinol proton δ_H_ 2.83 ppm (d, *J* = 4.3 Hz, 1 H) (H-6) in compound **4**, confirmed the successful epoxidation of stigmasterol. This was further confirmed by the disappearance of the alkene signals at 140.77 (C-5) and 121.73 (C-6) of the stigmasterol in the ^13^C NMR spectrum. The resonances at C-5 and C-6 were shifted upfield to δ_C_ 65.72 ppm and δ_C_ 59.30 ppm, respectively in the ^13^C NMR spectrum of compound **4** confirming the epoxy quaternary carbon resonances [39].

**5,6-Epoxystigmasta-3*β***,**22,23-triol (5)** was obtained as a white solid (0.7230 g, 1.5637 mmol, 66%) with a melting point of 82–85 °C. The specific optical rotation was recorded as [α]_D_^20.3^ = -51.7° (c = 1.50, CHCl_3_). The Q-TOF mass spectrum showed a molecular ion [M + Na]^+^ peak at *m/*z 486.3954 with the molecular formula of C_29_H_50_O_4_. The IR spectrum showed a broad (-OH) peak appearing at 3409.8 cm^− 1^, the absorption band at 2942.2 cm^− 1^ was attributed to CH_2_ stretching, the (C = O) stretch was observed at 1660.5 cm^− 1^ and the absorption bands at 1062.1 cm^− 1^ were due to a (C-O) stretching.

Compound **5** was confirmed by the appearance of oxymethine protons at δ_H_ 3.68 ppm and δ_H_ 3.48 ppm in the ^1^H NMR spectrum of the product which was seen corresponding to C-22 and C-23. The absence of the olefinic protons at δ_H_ 5.06 ppm (dd, *J* = 15.2, 8.5 Hz, 1 H) (H-22) and δ_H_ 4.93 ppm (dd, *J =* 15.2, 8.5 Hz, 1 H) (H-23) of compound **4** in the ^1^H NMR spectrum and the presence of the new oxymethine protons confirmed the successful addition of the hydroxyl groups at this position. This was further confirmed by the absence of olefinic carbon signals at δ_C_ 138.23 (C-22) and 129.34 (C-23), which were shifted upfield in the ^13^C NMR spectrum of compound **5** [39].

**Stigmastane-3*β***,**5,6,22,23-pentol (6)** was obtained as a colourless oil (0.533 g, 1.1095 mmol, 81%) with a melting point of 201–204 °C. The specific optical rotation was recorded as [α]_D_^20.1^ = -29.6° (c = 0.60, CHCl_3_). The Q-TOF mass spectrum showed a molecular ion [M-Na]^−^ peak at *m/*z 447.3738 with the molecular formula of C_29_H_52_O_5_. The IR spectrum showed a broad (-OH) peak appearing at 3416.3 cm^− 1^, the absorption band at 2939.0 was attributed to C-H (alky) stretching and the absorption band at 1045.8 cm^− 1^ was due to a (C-O) stretching.

The disappearance of the 2 olefinic protons at δ_H_ 5.06 ppm (dd, *J* = 15.2, 8.5 Hz, 1 H) (H-22)and δ_H_ 4.93 ppm (dd, *J =* 15.2, 8.5 Hz, 1 H) (H-23) of compound **4** and the appearance of 3 more oxymethine protons at δ_H_ 3.53 ppm, δ_H_ 2.73 ppm (dd, *J* = 7.2, 2.3 Hz, 1 H) and δ_H_ 2.49 ppm confirmed the successful opening of the epoxide ring in compound **6**. The new oxymethine protons were seen corresponding to δ_C_ 75.85 ppm (C-6), δ_C_ 63.10 ppm (C-22) and δ_C_ 62.12 ppm (C-23) respectively in the HSQC spectrum. The proton at δ_H_ 3.53 ppm (H-6) was seen correlating to C-4 and the proton at δ_H_ 2.73 ppm (dd, *J* = 7.2, 2.3 Hz, 1 H) (H-22) was seen correlating to C-20 and C-24 in the HMBC spectrum which confirmed the assignment of the hydroxyl groups at C-6, C-22 and C-23 [43].

**Stigmasta-5-en-3,7-dion-22,23-diol (7)** was obtained as a white powder (0.0947 g, 0.2131 mmol, 22%) with a melting point of 137–140 °C. The specific optical rotation was recorded as [α]_D_^20.1^ = -43.9° (c = 0.85, CHCl_3_). The Q-TOF mass spectrum showed a molecular ion [M + H]^+^ peak at *m/*z 459.3487 with the molecular formula of C_29_H_46_O_4_. The IR spectrum showed a broad (-OH) peak appearing at 3416.3 cm^− 1^, the absorption band at 2876.8 cm^− 1^ was attributed to C-H stretching, the (C = C) stretch was observed at 1693.2 cm^− 1^ and the absorption bands at 1735.7 cm^− 1^ were due to a (C = O) stretching.

The structure of compound **7** was confirmed by the absence of the oxymethine proton at δ_H_ 4.09 ppm (dd, *J* = 11.5, 6.3 Hz, 1 H) (H-3) of compound **6** and the appearance of a sharp and long singlet at δ_H_ 6.16 ppm attributed to an olefinic proton (H-6) in the ^1^H NMR spectrum of compound **7**. Furthermore, there was an appearance of signals at δ_C_ 199.35 and δ_C_ 202.06 ppm in the ^13^C NMR spectrum attributed to the keto carbonyl carbons C-3 and C-7, respectively. This is the first report of compound **7**.

**Stigmasta-3,7-dion-5,6,22,23-ol** (**8**) was obtained as a white solid (0.163 g, 0.3407 mmol, 36%) with a melting point of 183–187 °C. The specific optical rotation was recorded as [α]_D_^18.7^= -26.9° (c = 1.12, CHCl_3_). The Q-TOF mass spectrum showed a molecular ion [M + H]^+^ peak at *m/*z 495.3071 with the molecular formula of C_29_H_48_O_6_. The IR spectrum showed a broad (-OH) peak appearing at 3364.0 cm^− 1^, the absorption band at 2873.6 cm^− 1^ was attributed to C-H stretching and the (C = O) stretch was observed at 1709.5 cm^− 1^.

The ^1^H NMR spectrum of compound **8** showed a shift in the proton resonance of H-6 from δ_H_ 3.53 ppm to δ_H_ 3.16 ppm confirming the addition of a hydroxyl group at C-6. The absence of the oxymethine proton resonance at δ_H_ 4.09 ppm (dd, *J* = 11.5, 6.3 Hz, 1 H) (H-3) of compound **6**, confirmed the introduction of a carbonyl at position C-3 in compound **8**. Furthermore, there was an appearance of signals at δ_C_ 211.07 ppm and δ_C_ 211.33 ppm in the ^13^C NMR spectrum of compound **8** attributed to the keto carbonyl carbons C-3 and C-7. This is the first report of compound **8**.

**Stigmast-5-ene-3*β***,**22,23-triol** (**9**) was obtained as a white solid (0.102 g, 0.2285 mmol, 24%) with a melting point of 181–183 °C. The specific optical rotation was recorded as [α]_D_^19.5^= -6.5° (c = 1.81, CHCl_3_). The Q-TOF mass spectrum showed a molecular ion [M-H] ^−^ peak at *m/*z 445.3581 with the molecular formula of C_29_H_50_O_3_. The IR spectrum showed a broad (-OH) peak appearing at 3403.3, the absorption band at 2870.3 cm^− 1^ was attributed to C-H stretching and the (C = C) stretch was observed at 1683.4 cm^− 1^.

The ^1^H NMR spectrum of compound **9** indicated that the oxymethine proton at δ_H_ 3.53 ppm (H-6) of compound **6** changed to an olefinic proton δ_H_ 5.80 ppm (H-6). There was also an appearance of olefinic carbon resonances at δ_C_ 168.43 ppm (C-5) and δ_C_ 126.30 ppm (C-6) in the ^13^C NMR spectrum [39].

### Cytotoxic activities of stigmasterol derivatives

Stigmasterol and all synthesized stigmasterol derivatives were screened in vitro against breast cancer cells including, the triple-negative breast cancer (HCC70), hormone receptor-positive breast cancer (MCF-7) and non-tumorigenic epithelial cell lines established from breast tissue (MCF-12 A) cell lines. Camptothecin was used as the positive control. The cytotoxicity data for all the synthetic derivatives are summarized in Table 1.

The synthesized stigmasterol analogues showed improved cytotoxic activity compared to the stigmasterol (**1**), which was not toxic to the cancer cell lines (EC_50_ ˃ 250 µM). Overall, the stigmasterol derivatives [stigmasterol acetate (**2**), stigmasta-5,22-dien-3,7-dione (**3**), 5,6-epoxystigmast-22-en-3*β*-ol (**4**), 5,6-epoxystigmasta-3*β*,22,23-triol (**5**), stigmastane-3*β*,5,6,22,23-pentol (**6**), stigmasta-5-en-3,7-dion-22,23-diol (**7**), stigmasta-3,7-dion-5,6,22,23-ol (**8**) and stigmast-5-ene-3*β*,22,23-triol (**9**)], demonstrated either modest toxicity [for example, stigmasta-5,22-dien-3,7-dione (**3**): 42.85 ± 1.32 and 67.73 ± 1.26 against MCF-7 and HCC70 breast cancer cell lines] or were found to be nontoxic [for example, stigmasterol acetate (**2**): EC_50_ > 250 µM) against all three of the cell lines] as illustrated in Table 1. The exceptions were 5,6-epoxystigmast-22-en-3*β*-ol (**4**), stigmastane-3*β*,5,6,22,23-pentol (**6**) and stigmast-5-ene-3*β*,22,23-triol (**9**), which showed reasonable cytotoxic activity against MCF-7 and HCC70 cell lines. 5,6-epoxystigmast-22-en-3*β*-ol (**4**) and stigmast-5-ene-3*β*,22,23-triol (**9**), displayed EC_50_ values of 21.92 and 22.94 µM, respectively against hormone responsive MCF-7 breast cancer cells, while stigmastane-3*β*,5,6,22,23-pentol (**6**) exhibited an EC_50_ value of 16.82 µM against the triple negative breast cancer HCC70 cell line. In terms of selectivity of the compounds for the latter cancer cell lines over the non-cancerous control cell line, only 5,6-epoxystigmast-22-en-3*β*-ol (**4**) and stigmast-5-ene-3*β*,22,23-triol (**9**) display selectivity indices above 2, indicating that these compounds are more toxic to cancer cells (in particular MCF-7 cells) versus non-cancerous cells. The role of the epoxide ring and the hydroxyl group differed among the cell types. The presence of an epoxide ring seemed to enhance the activity in studies against the hormone responsive MCF-7 breast cancer cells. An increase in the number of hydroxyl groups appeared to increase the cytotoxic activity of the derivatives when the derivatives were screened against the triple negative breast cancer HCC70 cell line. This is the first report on breast cancer studies of these stigmasterol derivatives.

**Table 1:** In vitro cytotoxicity results of the synthesized stigmasterol derivatives against MCF-12 A, MCF-7 and HCC70 breast cells

Compound name	MCF-12 A		MCF-7		HCC70	
	EC_50_ and Standard deviation (**µM**)	**R** ^**2**^	EC_50_ and Standard deviation (**µM**)	**R** ^**2**^	EC_50_ and Standard deviation (**µM**)	**R** ^**2**^
stigmasterol (**1**)	NT		NT		NT	
stigmasterol acetate (**2**)	NT		NT		NT	
stigmasta-5,22-dien-3,7-dione (**3**)	30.18 ± 1.24	0.93	42.85 ± 1.32 **SI**: 0.70	0.88	67.73 ± 1.26 **SI**: 0.45	0.91
5,6-epoxystigmast-22-en-3*β*-ol (**4**)	46.97 ± 1.18	0.94	21.92 ± 1.32 **SI**: 2.14	0.82	165.70 ± 1.30 **SI**: 0.28	0.85
5,6-epoxystigmasta-3*β*,22,23-triol (**5**)	30.18 ± 1.28	0.90	43.99 ± 1.40 **SI**: 0.69	0.84	33.69 ± 1.33 **SI**: 0.90	0.88
stigmastane-3*β*,5,6,22,23-pentol (**6**)	22.56 ± 1.22	0.93	44.14 ± 1.40 **SI**: 0.51	0.84	16.82 ± 1.17 **SI**: 1.34	0.96
stigmasta-5-en-3,7-dion-22,23-diol (**7**)	31.60 ± 1.12	0.93	23.16 ± 1.08 **SI**:1.36	0.95	170.10 ± 1.18 **SI**:0.19	0.80
stigmasta-3,7-dion-5,6,22,23-ol (**8**)	NT		NT		53.23 ± 1.06	0.97
stigmast-5-ene-3*β*,22,23-triol (**9**)	51.79 ± 1.13	0.92	22.94 ± 1.09 **SI**:2.26	0.94	44.54 ± 1.13 **SI**:1.16	0.91
DCM crude (*R. tridentate)*	96.37 ± 1.19	0.87	˃250		13.82 ± 1.12 **SI**: 6.97	0.87
**Camptothecin** (**nM**)	104.20 ± 1.03	0.99	103.80 ± 9.92 **SI**:1.00	0.99	83.17 ± 1.08 **SI**:1.25	0.99

Key: NT = not toxic (EC_50_ > 250 µM); SI = selectivity index

## Conclusion

Eight derivatives (**2**–**9**) of stigmasterol (**1**) were successfully synthesized via acetylation, oxidation, epoxidation, ring-opening and dihydroxylation of the epoxide. The structures of all the compounds were determined using spectroscopic techniques and melting points. Stigmasta-5-en-3,7-dion-22,23-diol (**7**) and Stigmasta-3,7-dion-5,6,22,23-ol (**8**) are reported for the first time. Stigmasterol and all the synthesized derivatives were screened against non-tumorigenic epithelial cell lines established from breast tissue (MCF-12 A), hormone receptor-positive breast cancer (MCF-7) and triple-negative breast cancer (HCC70) cell lines. The synthesized stigmasterol analogues demonstrated increased cytotoxic activity overall compared to the stigmasterol (**1**), which was not toxic to the cancer cell lines (EC_50_ ˃ 250 µM). In particular, 5,6-epoxystigmast-22-en-3*β*-ol (**4**) and stigmast-5-ene-3*β*,22,23-triol (**9**) showed improved cytotoxic activity and a degree of selectivity against MCF-7 breast cancer cells, with EC_50_ values of 21.92 and 22.94 µM, respectively, and selectivity indices of 2.14 and 2.26, respectively. On the other hand, stigmastane-3*β*,5,6,22,23-pentol (**6**) demonstrated increased cytotoxicity against the triple negative breast cancer HCC70 cell line, with an EC_50_ value of 16.82 µM, although this compound was not selective. Structural modification has proven to be a valid strategy to increase the anticancer activity of stigmasterol since all the synthesized derivatives displayed greatly improved activity. This represents a solid starting point in the development of novel anticancer drugs. Stigmasterol derivatives have potential as candidates for novel anticancer drugs hence further study of these compounds is needed.

### Electronic supplementary material

Below is the link to the electronic supplementary material.

Supplementary Material 1

## Data Availability

All data generated or analysed during this study are included in this published article [and its supplementary information files].
