# Supplementary material for: In vitro cytotoxic effect of stigmasterol derivatives against breast cancer cells
Source: BMC Complement Med Ther. 2023 Sep 11;23:316. doi: 10.1186/s12906-023-04137-y (PMC10496295; doi:10.1186/s12906-023-04137-y)
Supplement: Supplementary file 1 — Supplementary Material 1 [file 12906_2023_4137_MOESM1_ESM.docx]

***In vitro* cytotoxic effect of Stigmasterol derivatives against breast cancer cells**

Nondumiso Premilla Dube ^a^, Vuyelwa Jacqueline Tembu ^a^, Getrude R Nyemba ^b^, Candace Davison ^b^, Goitsemodimo Herckious Rakodi ^a^, Douglas Kemboi ^a,d^, Jo-Anne de la Mare ^b^, Xavier Siwe-Noundou ^c^, Amanda Ezra-Lee Manicum ^a*^

*^a^ Department of Chemistry, Tshwane University of Technology, Private Bag X680, Pretoria 0001, South Africa*

*^b^ Department of Biochemistry and Microbiology, Female Cancers Research at Rhodes University (FemCR_2_U), Makhanda/Grahamstown, 6140, South Africa*

*^c^ Department of Pharmaceutical Science, Sefako Makgatho Health Science University, Pretoria 0204, South Africa,*

*^d^Department of Physical Sciences, University of Kabianga, Kericho, 2030, Kenya.*

* Corresponding authors. E-mail address: [TembuVJ@tut.ac.za](mailto:TembuVJ@tut.ac.za) (V.J. Tembu)

[ManicumAE@tut.ac.za](mailto:ManicumAE@tut.ac.za) (A. E. Manicum).

**Abstract**

**Background:** Stigmasterol is an unsaturated phytosterol that belongs to the class of tetracyclic steroids abundant in various medicinal plants. Stigmasterol is an important constituent since it has been proven to possess impressive pharma­cological effects such as anti-osteoarthritis, anticancer, anti-diabetic, anti-inflammatory, antiparasitic, immunomodulatory, antifungal, antioxidant, antibacterial, and neuroprotective activities. Furthermore, due to the presence of *π* system and hydroxyl group, stigmasterol is readily derivatized through substitution and addition reactions, allowing for the synthesis of a wide variety of stigmasterol derivatives.

**Methods:** Stigmasterol (**1**) was used as starting material to form eight bio-active derivatives (**2**-**9**) through acetylation, epoxidation, epoxide ring opening, oxidation, and dihydroxylation reactions. The structures of all the compounds were established using spectroscopic techniques, NMR, IR, MS, and melting points. The synthesized stigmasterol derivatives were screened for cytotoxicity against the hormone receptor-positive breast cancer (MCF-7), triple-negative breast cancer (HCC70), and non-tumorigenic mammary epithelial (MCF-12A) cell lines using the resazurin assay.

**Results:** Eight stigmasterol derivatives were successfully synthesized namely; Stigmasterol acetate (**2**), Stigmasta-5,22-dien-3,7-dione (**3**), 5,6-Epoxystigmast-22-en-3*β*-ol (**4**), 5,6-Epoxystigmasta-3β,22,23-triol (**5**), Stigmastane-3*β*,5,6,22,23-pentol (**6**), Stigmasta-5-en-3,7-dion-22,23-diol (**7**), Stigmasta-3,7-dion-5,6,22,23-ol (**8**) and Stigmast-5-ene-3*β*,22,23-triol (**9**). This is the first report of Stigmasta-5-en-3,7-dion-22,23-diol (**7**) and Stigmasta-3,7-dion-5,6,22,23-ol (**8**). The synthesized stigmasterol analogues showed improved cytotoxic activity overall compared to the 3*β*-stigmasterol (**1**), which was not toxic to the three cell lines tested (EC_50_ ˃ 250 µM). In particular, 5,6-Epoxystigmast-22-en-3*β*-ol (**4**) and stigmast-5-ene-3*β*,22,23-triol (**9**) displayed improved cytotoxicity and selectivity against MCF-7 breast cancer cells (EC_50_ values of 21.92 and 22.94 µM, respectively), while stigmastane-3*β*,5,6,22,23-pentol (**6**) showed improved cytotoxic activity against the HCC70 cell line (EC_50_: 16.82 µM).

**Conclusion:** Natural products and their derivatives exhibit a wide range of pharmacological activities, including anticancer activity. The results obtained from this study indicate that molecular modification of stigmasterol functional groups can generate structural analogues with improved anticancer activity. Stigmasterol derivatives have potential as candidates for novel anticancer drugs.

**Keywords**: Stigmasterol; Cytotoxicity; MCF-7; HCC70; MCF-12A.

**Appendix 1:** IR spectrum of Stigmasterol (**1**)

**
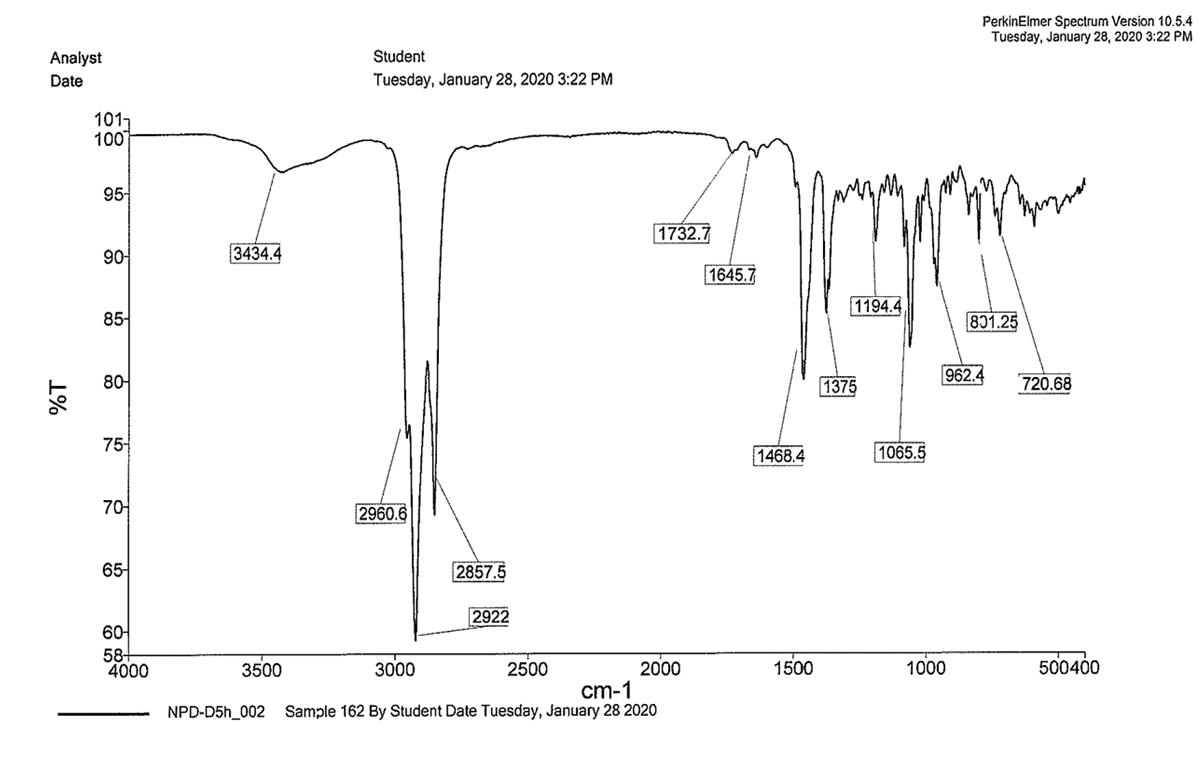
**

**Appendix 2:** MS spectrum of Stigmasterol (**1**)

**
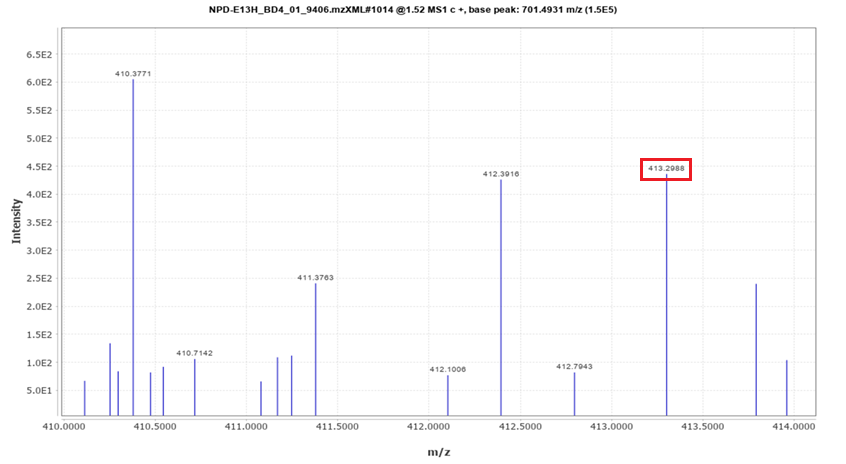
**

**Appendix 3:** ^1^H NMR spectrum of Stigmasterol (**1**) in CDCl_3_

**
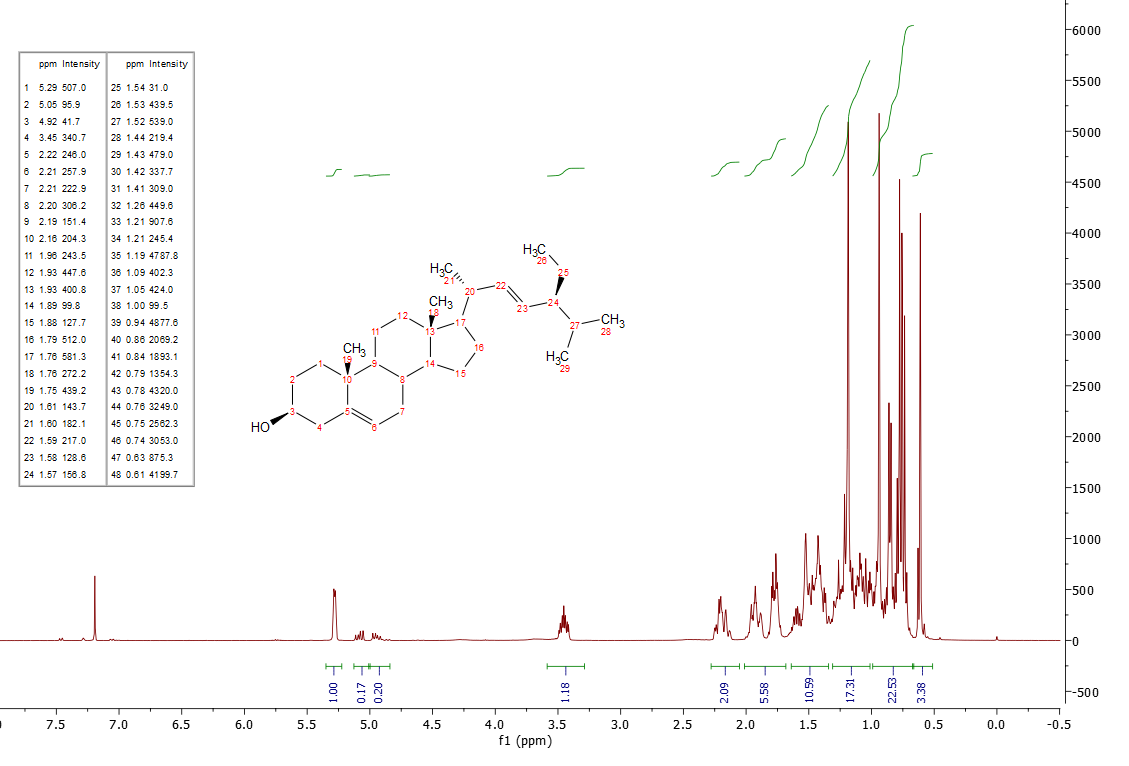
**

**Appendix 4:** ^13^C NMR spectrum of Stigmasterol (**1**) in CDCl_3_

**
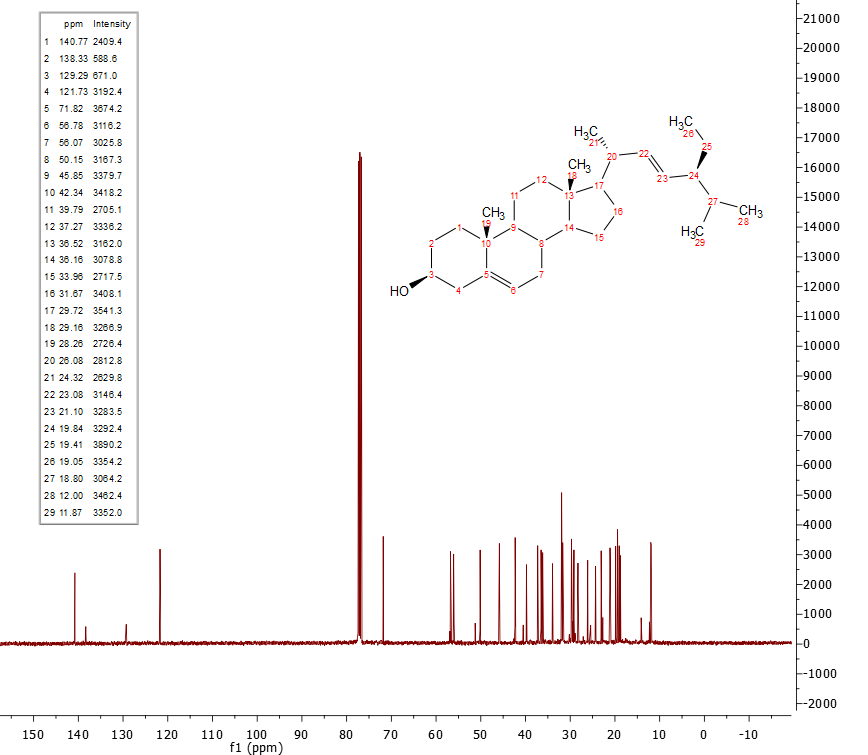
**

**Appendix 5:** DEPT spectrum of Stigmasterol (**1**)

**
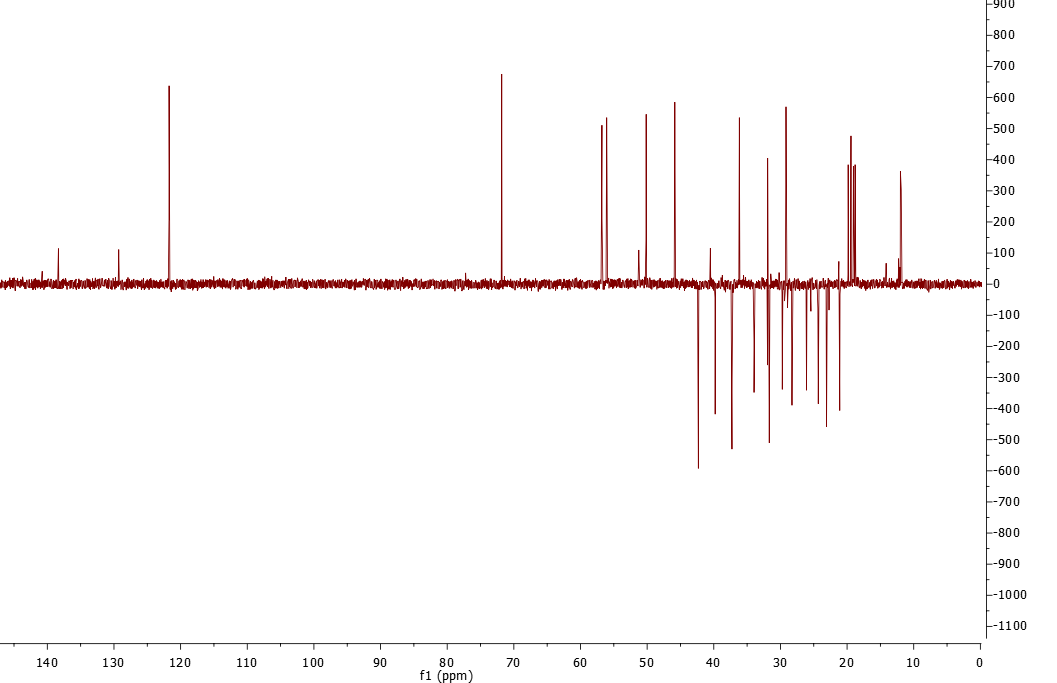
**

**Appendix 6:** HSQC spectrum of Stigmasterol (**1**)

**
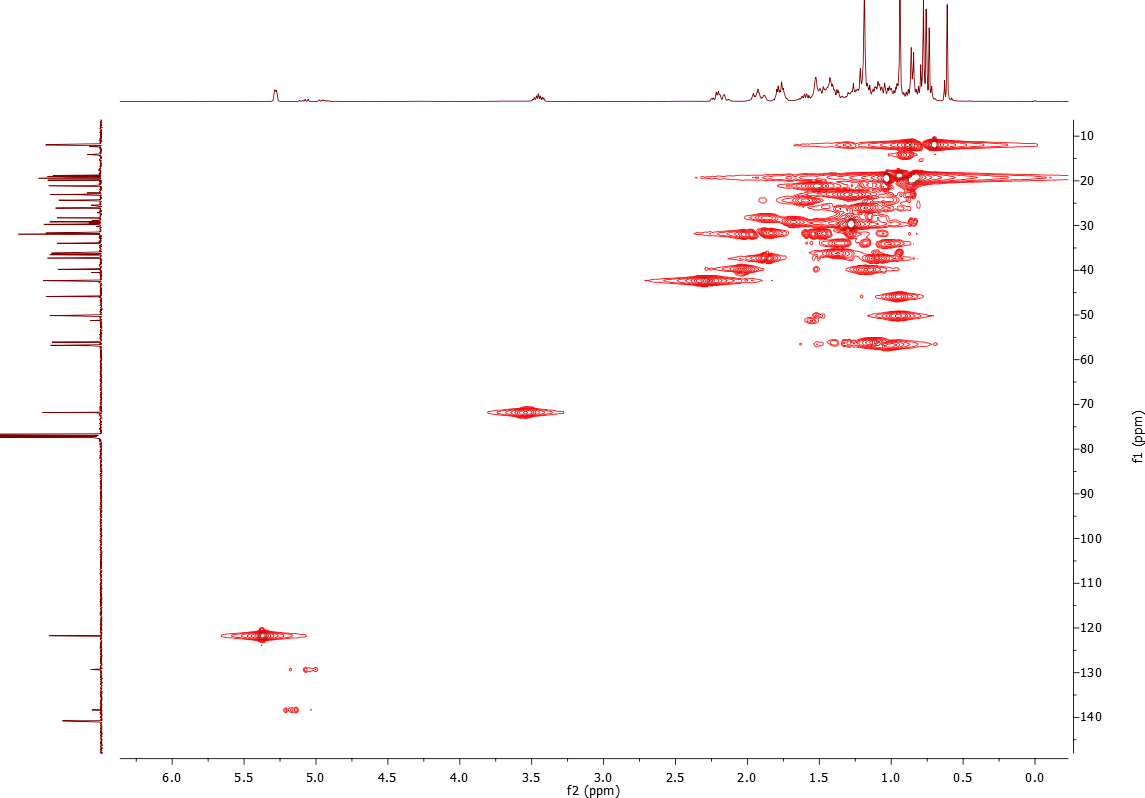
**

**Appendix 7:** HMBC spectrum of Stigmasterol (**1**)

**
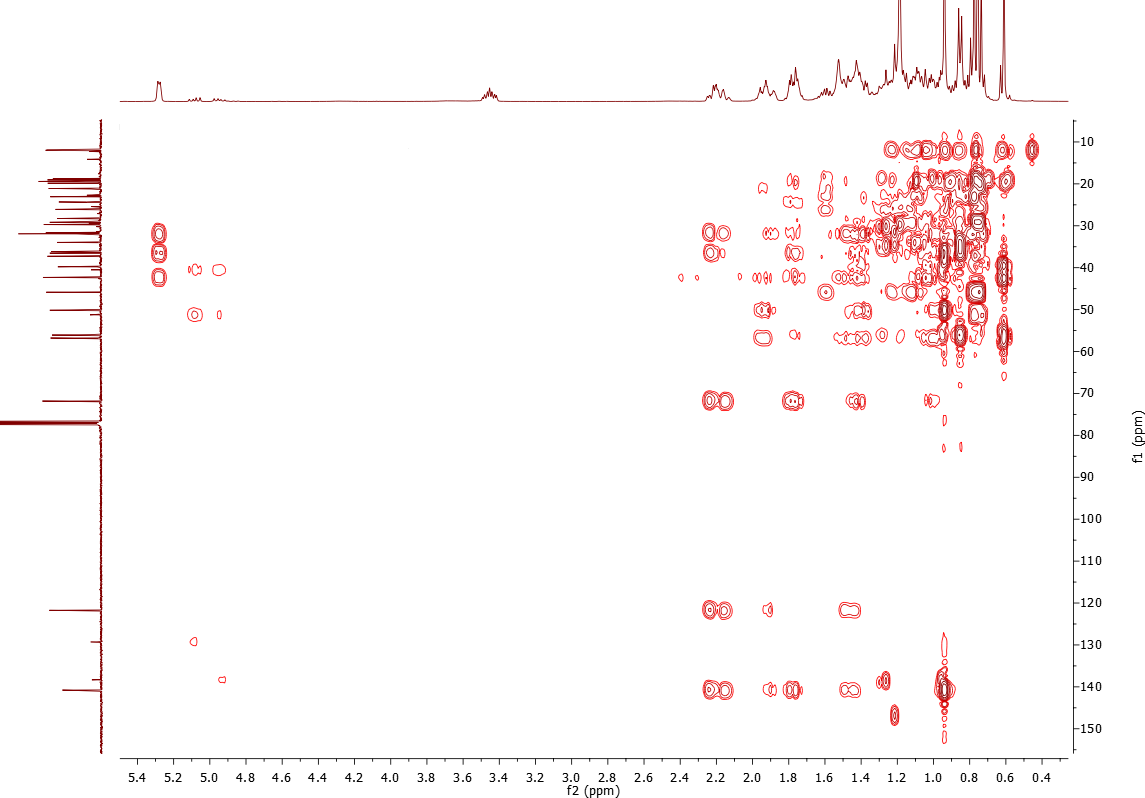
**

**Appendix 8:** COSY spectrum of Stigmasterol (**1**)

**
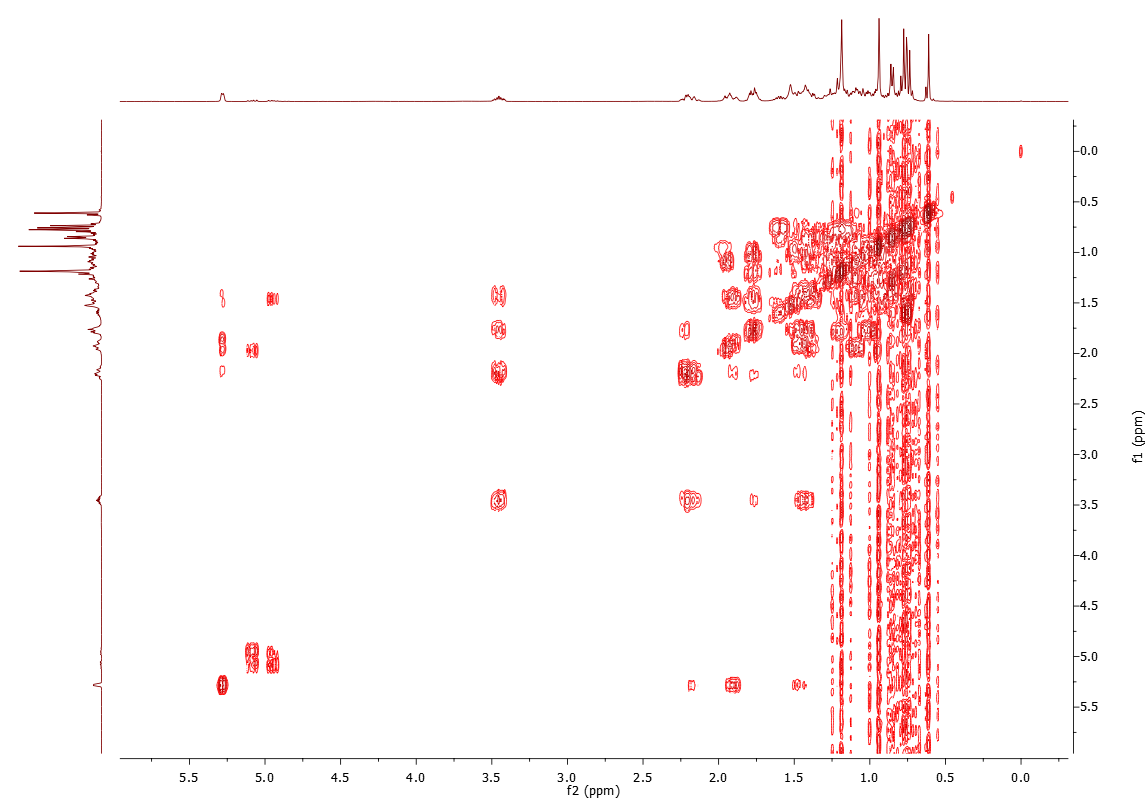
**

**Appendix 9:** NOESY spectrum of Stigmasterol (**1**)

**
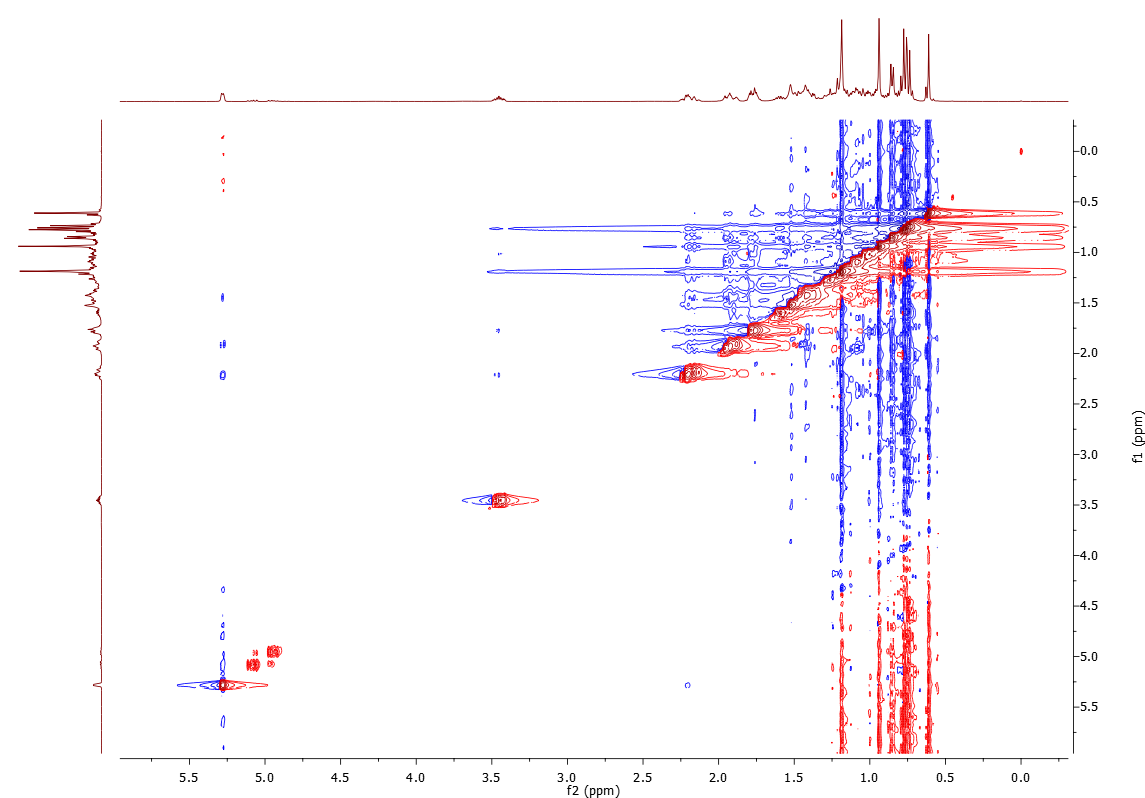
**

**Appendix 10:** IR spectrum of Stigmasterol acetate (**2**)

**
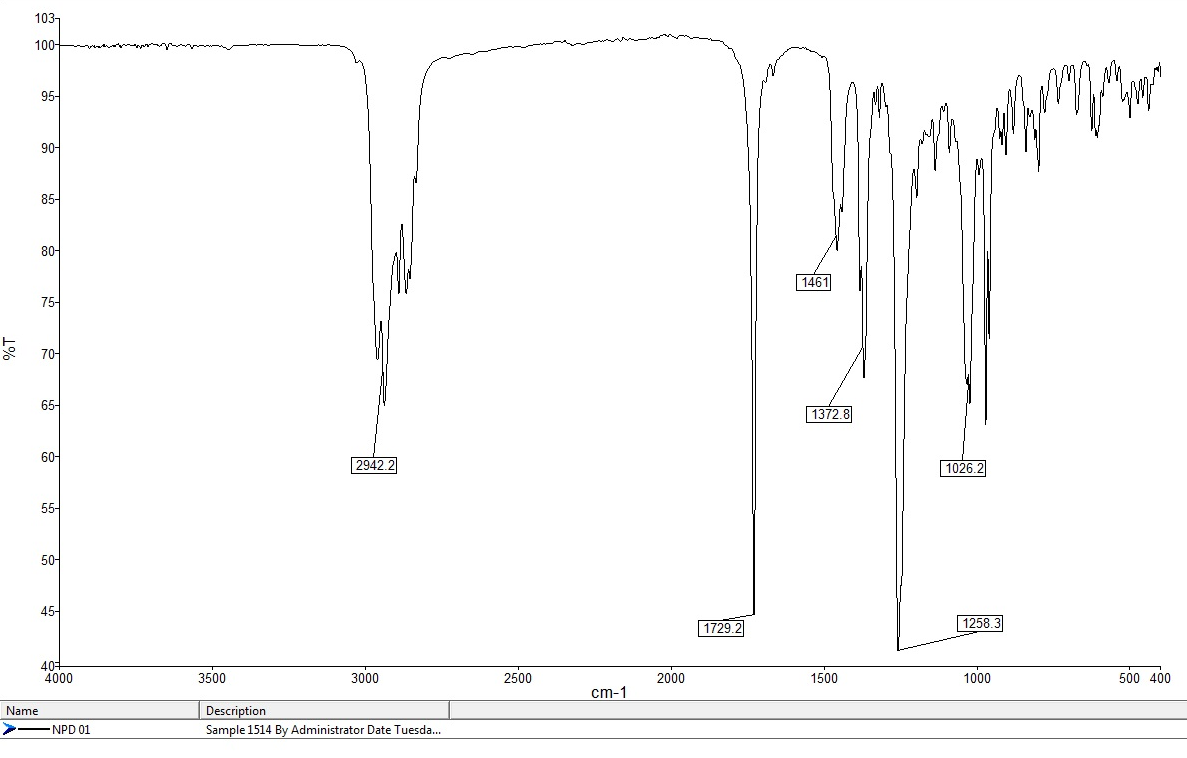
**

**Appendix 11:** MS spectrum of Stigmasterol acetate (**2**)

**
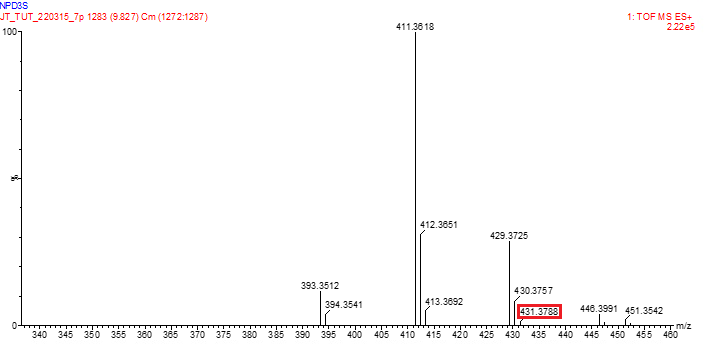
**

**Appendix 12:** ^1^H NMR spectrum of Stigmasterol acetate (**2**) in CDCl_3_

**
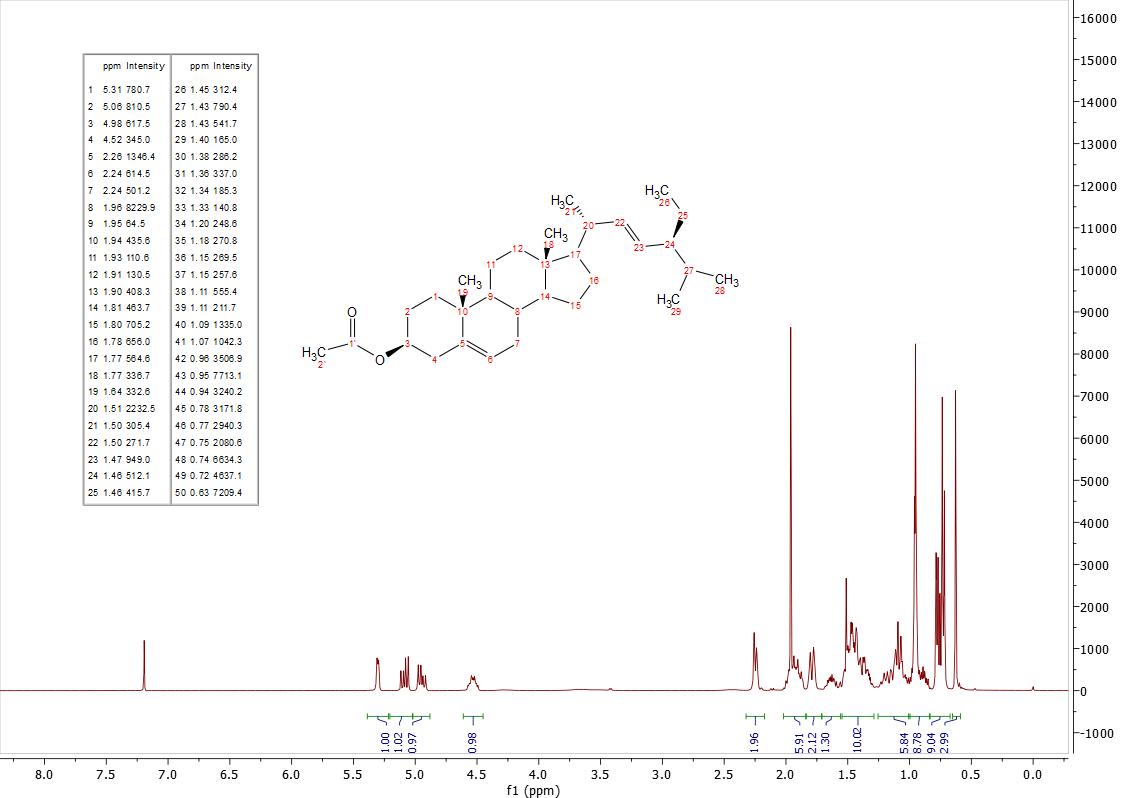
**

**Appendix 13:** ^13^C NMR spectrum of Stigmasterol acetate (**2**) in CDCl_3_

**
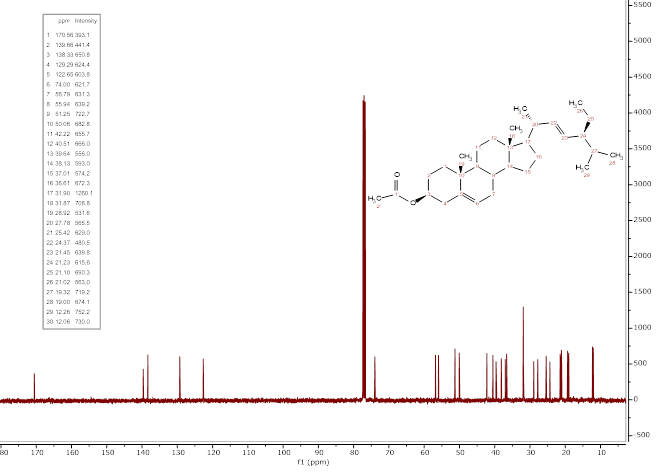
**

**Appendix 14:** IR spectrum of Stigmasta-5,22-dien-3,7-dione (**3**)

**
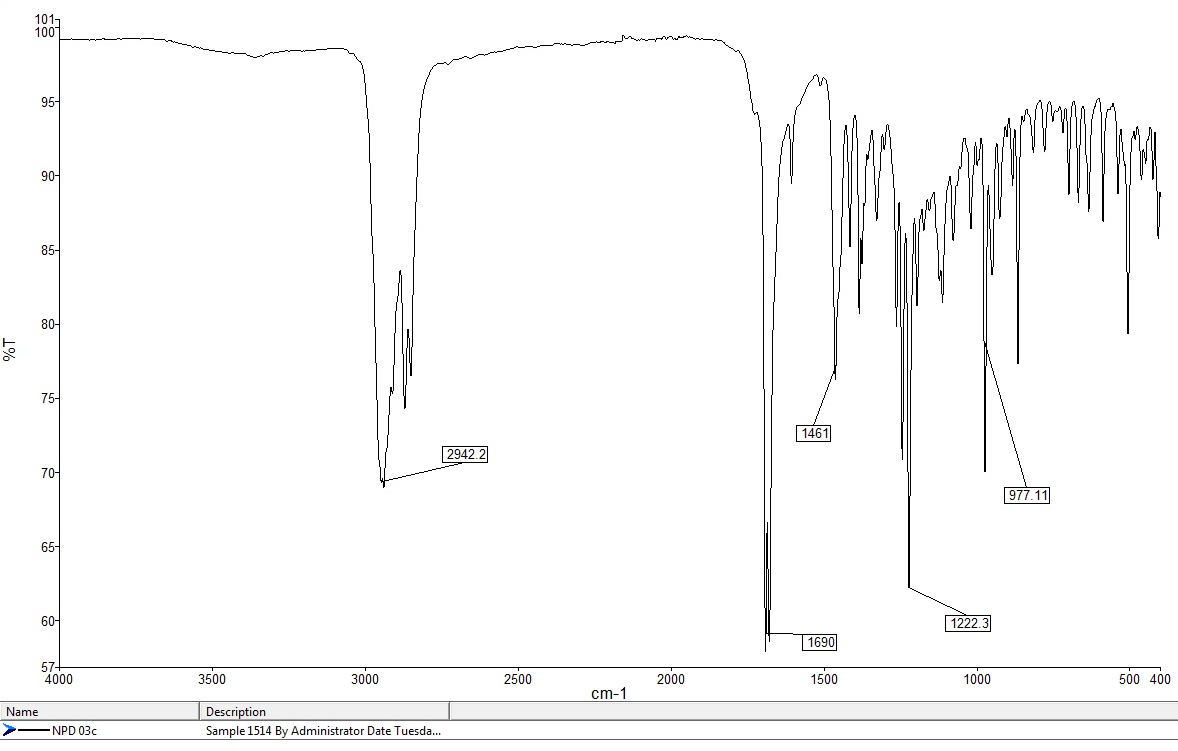
**

**Appendix 15:** MS spectrum of Stigmasta-5,22-dien-3,7-dione (**3**)

**
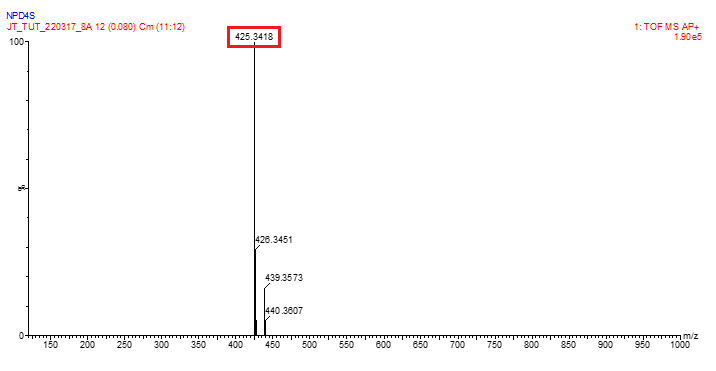
**

**Appendix 16:** ^1^H NMR spectrum of Stigmasta-5,22-dien-3,7-dione (**3**) in CDCl_3_

**
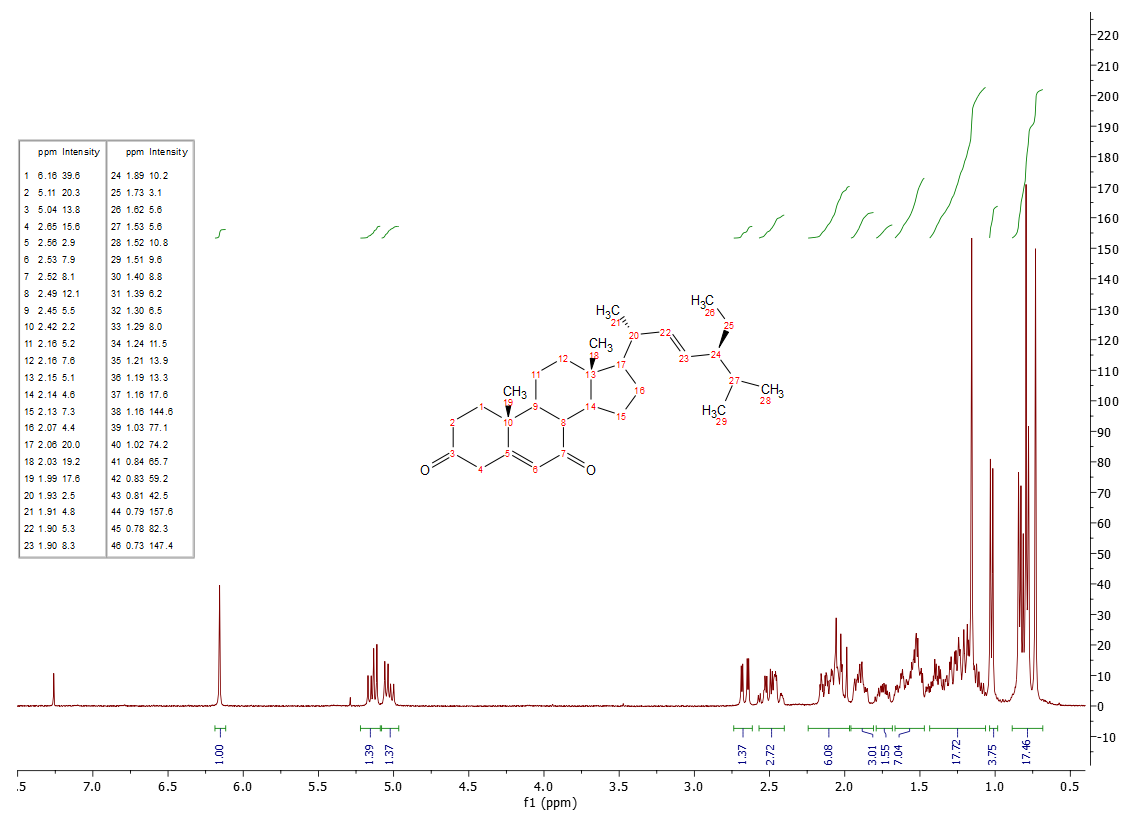
**

**Appendix 17:** ^13^C NMR spectrum of Stigmasta-5,22-dien-3,7-dione (**3**) in CDCl_3_

**
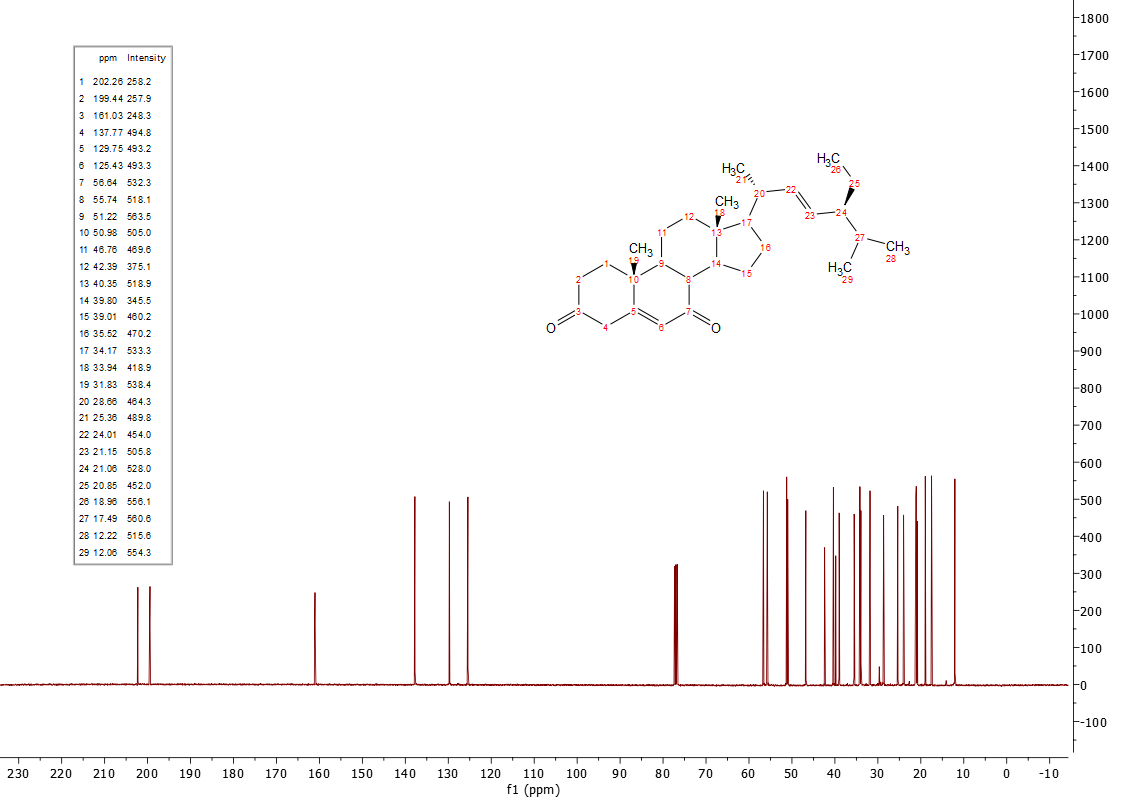
**

**Appendix 18:** DEPT spectrum of Stigmasta-5,22-dien-3,7-dione (**3**)

**
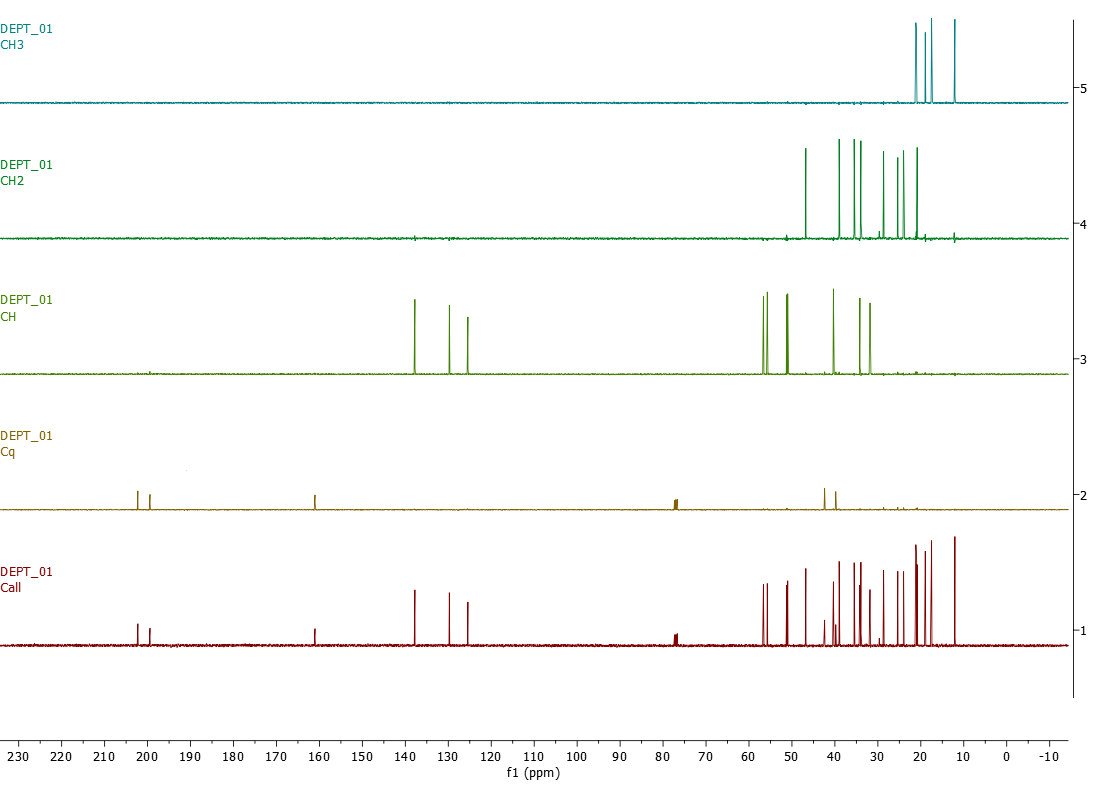
**

**Appendix 19:** HSQC spectrum of Stigmasta-5,22-dien-3,7-dione (**3**)

**
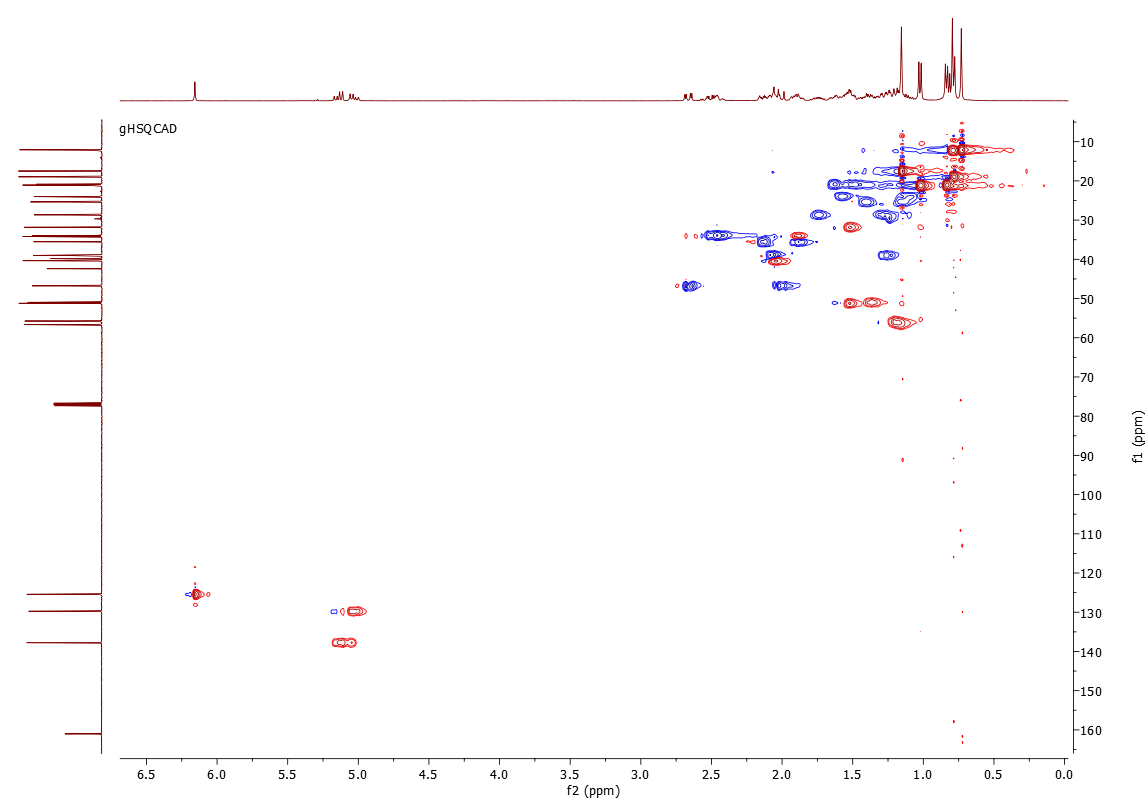
**

**Appendix 20:** HMBC spectrum of Stigmasta-5,22-dien-3,7-dione (**3**)

**
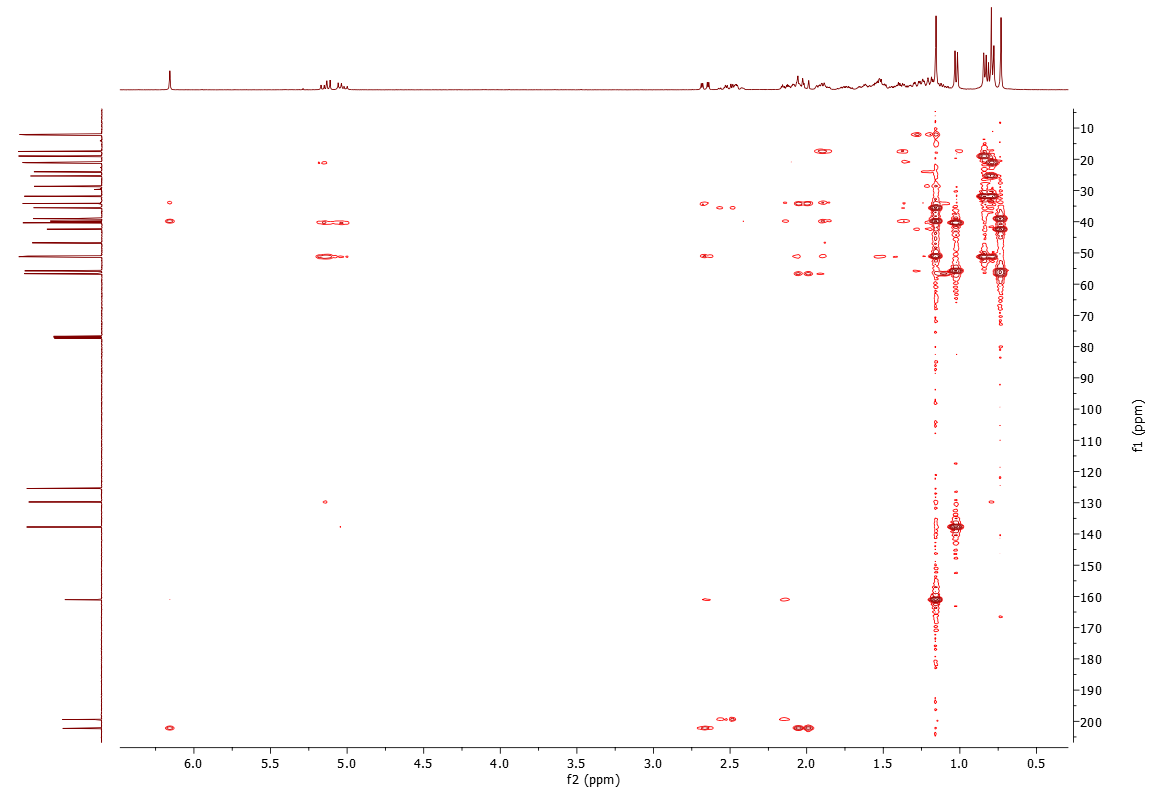
**

**Appendix 21:** COSY spectrum of Stigmasta-5,22-dien-3,7-dione (**3**)

**
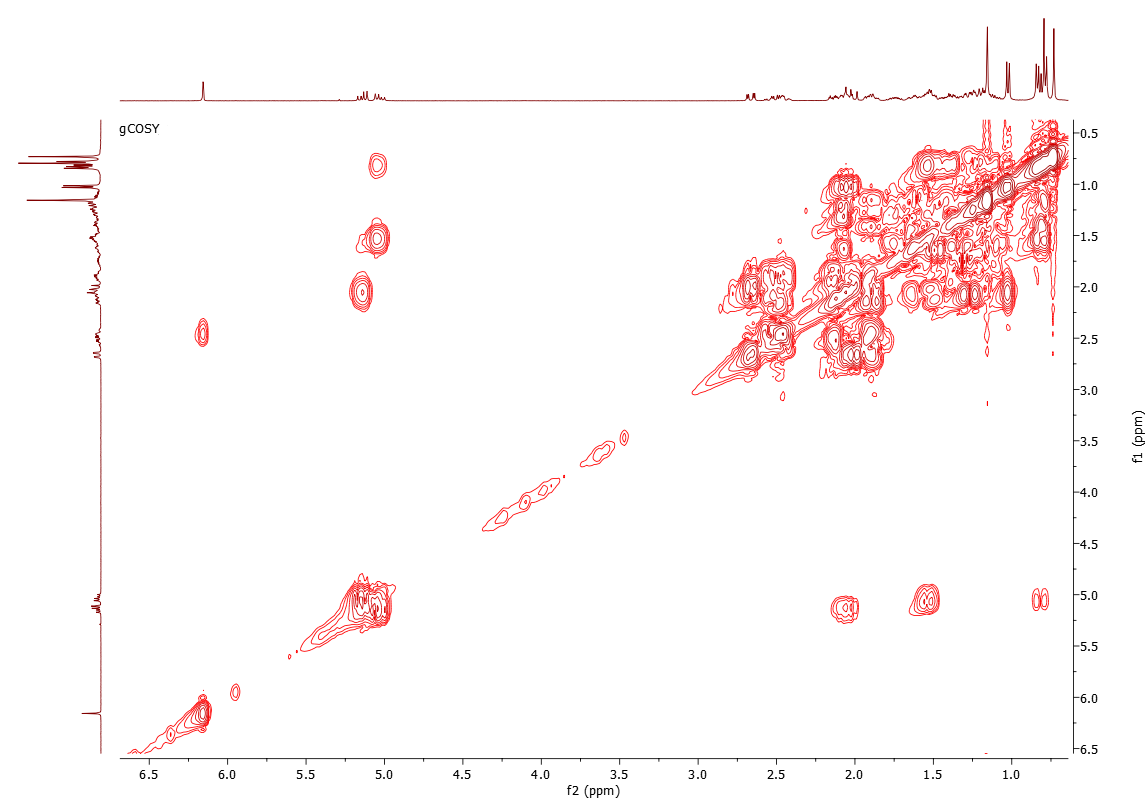
**

**Appendix 22:** IR spectrum of 5,6-Epoxystigmast-22-en-3*β*-ol (**4**)

**
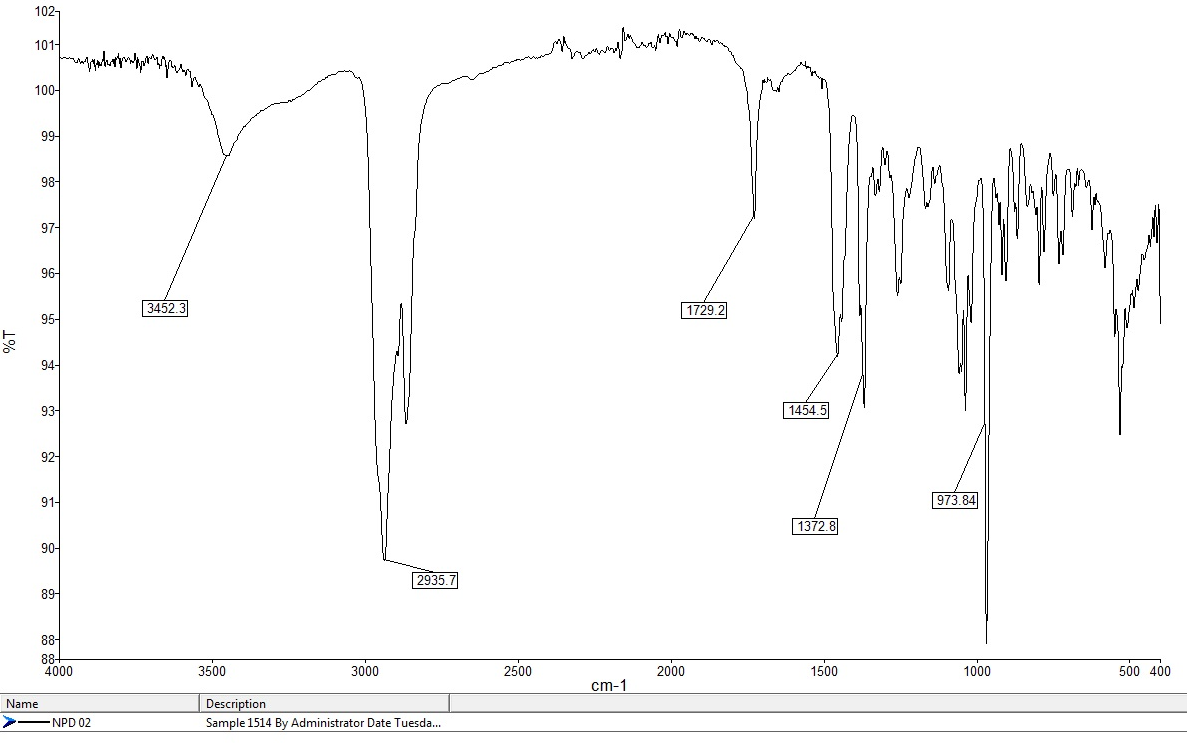
**

**Appendix 23:** MS spectrum of 5,6-Epoxystigmast-22-en-3*β*-ol (**4**)

**
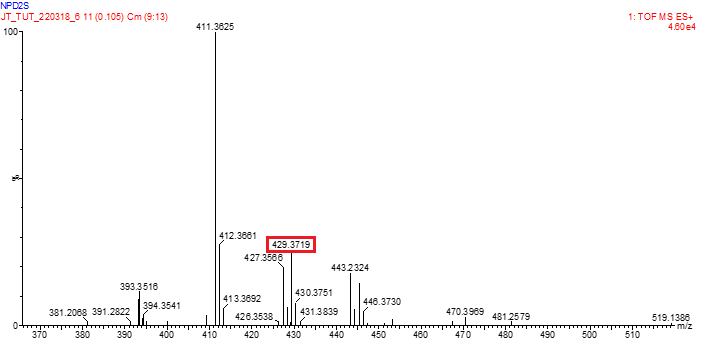
**

**Appendix 24:** ^1^H NMR spectrum of 5,6-Epoxystigmast-22-en-3*β*-ol (**4**) in CDCl_3_

**
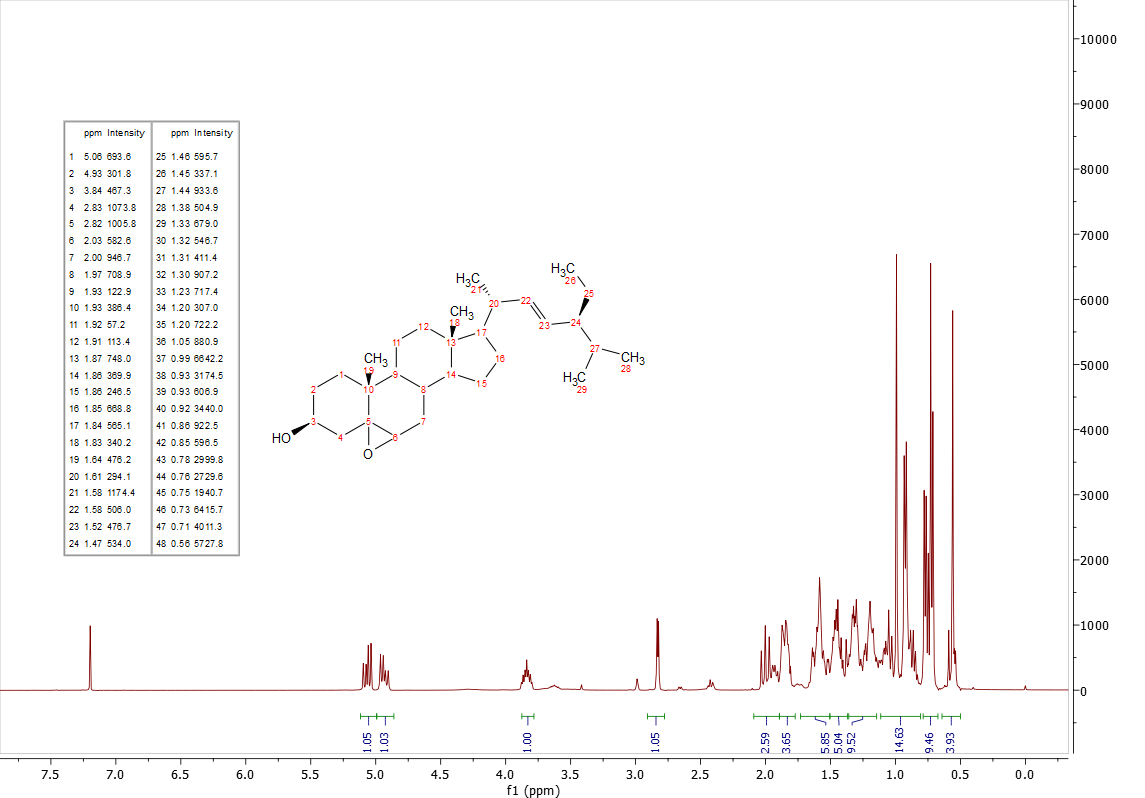
**

**Appendix 25:** ^13^C NMR spectrum of 5,6-Epoxystigmast-22-en-3*β*-ol (**4**) in CDCl_3_

**
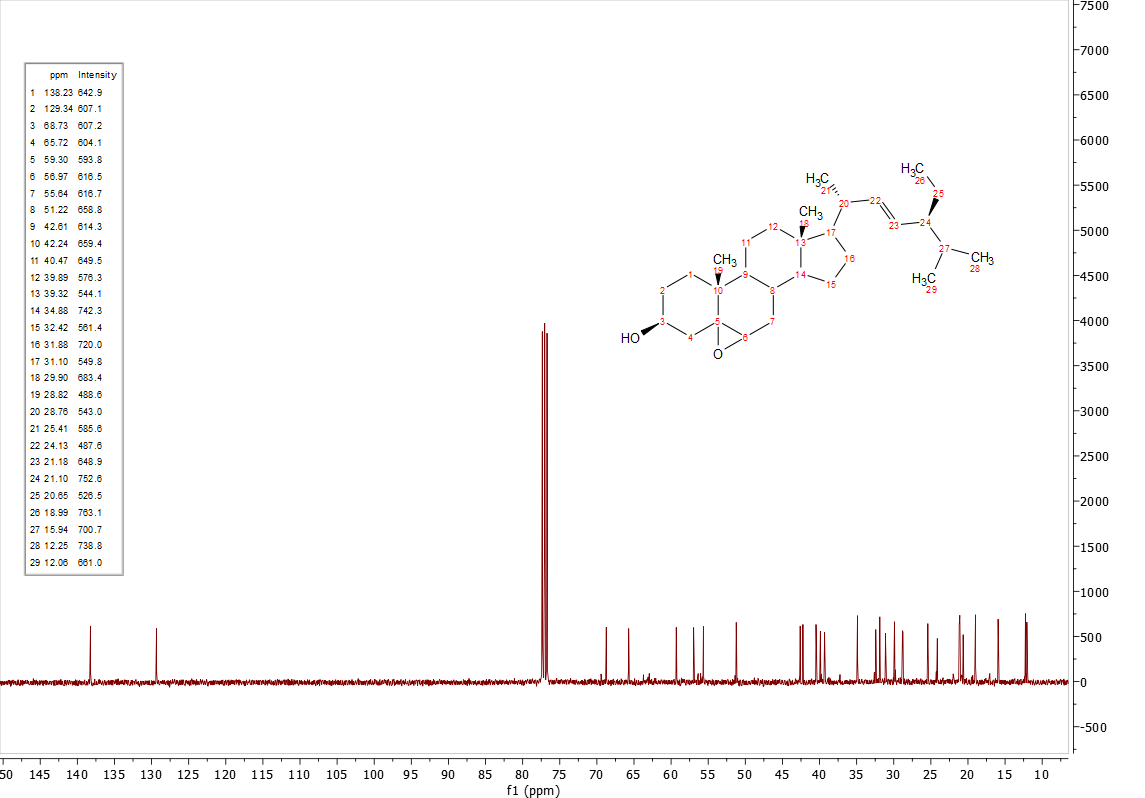
**

**Appendix 26:** IR spectrum of 5,6-Epoxystigmasta-3β,22,23-triol (**5**)

**
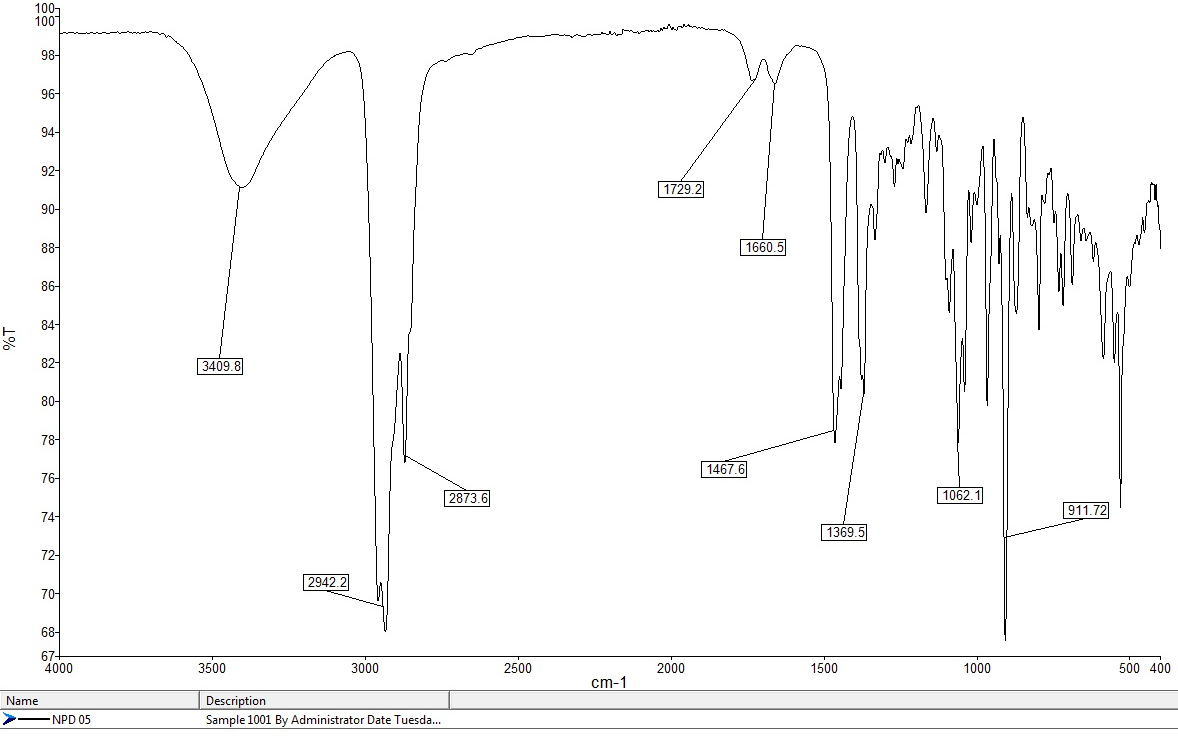
**

**Appendix 27:** MS spectrum of 5,6-Epoxystigmasta-3β,22,23-triol (**5**)

**
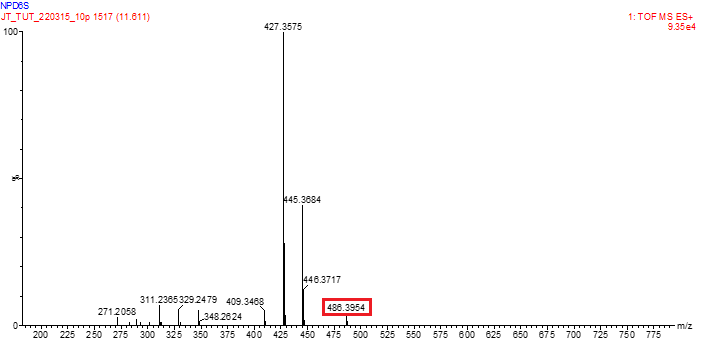
**

**Appendix 28:** ^1^H NMR spectrum of 5,6-Epoxystigmasta-3β,22,23-triol (**5**) in CDCl_3_

**
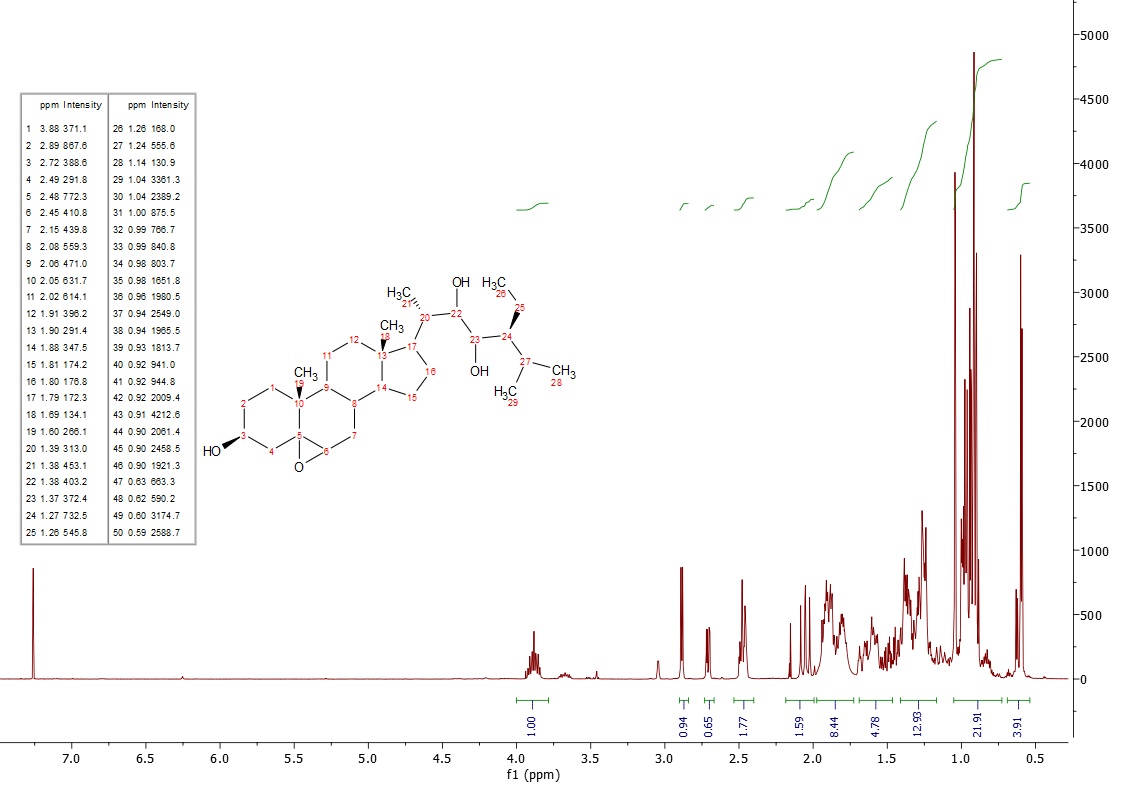
**

**Appendix 29:** ^13^C NMR spectrum of 5,6-Epoxystigmasta-3β,22,23-triol (**5**) in CDCl_3_

**
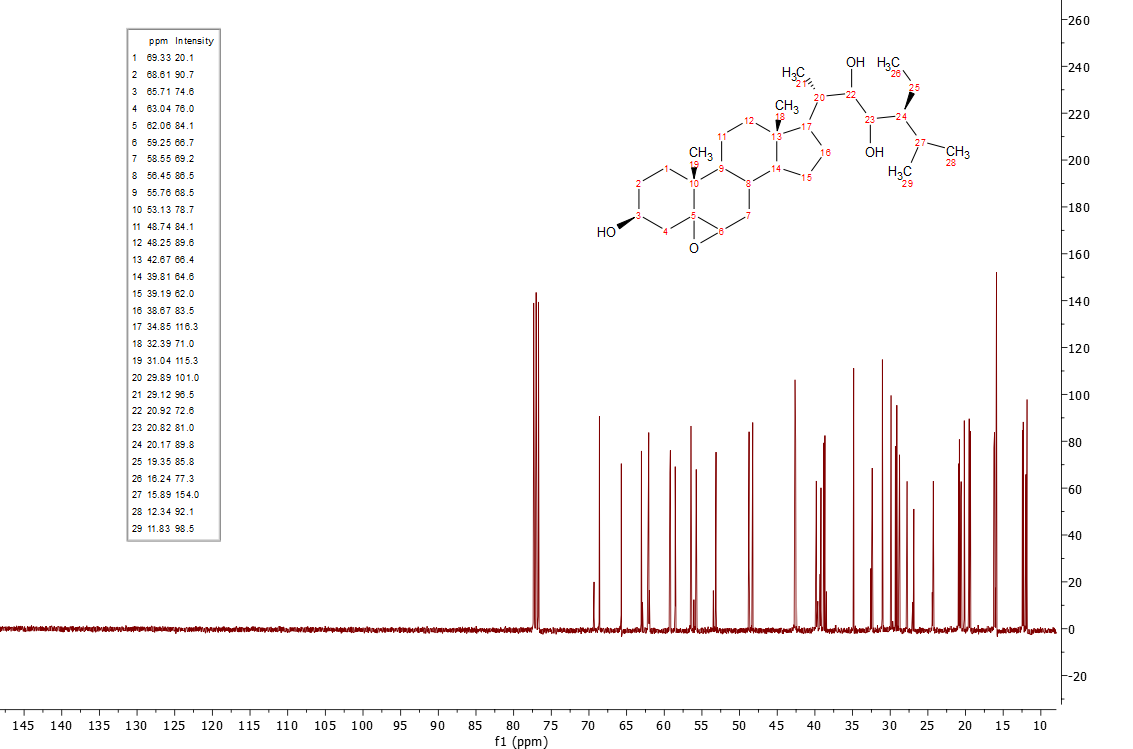
**

**Appendix 30:** HSQC spectrum of 5,6-Epoxystigmasta-3β,22,23-triol (**5**)

**
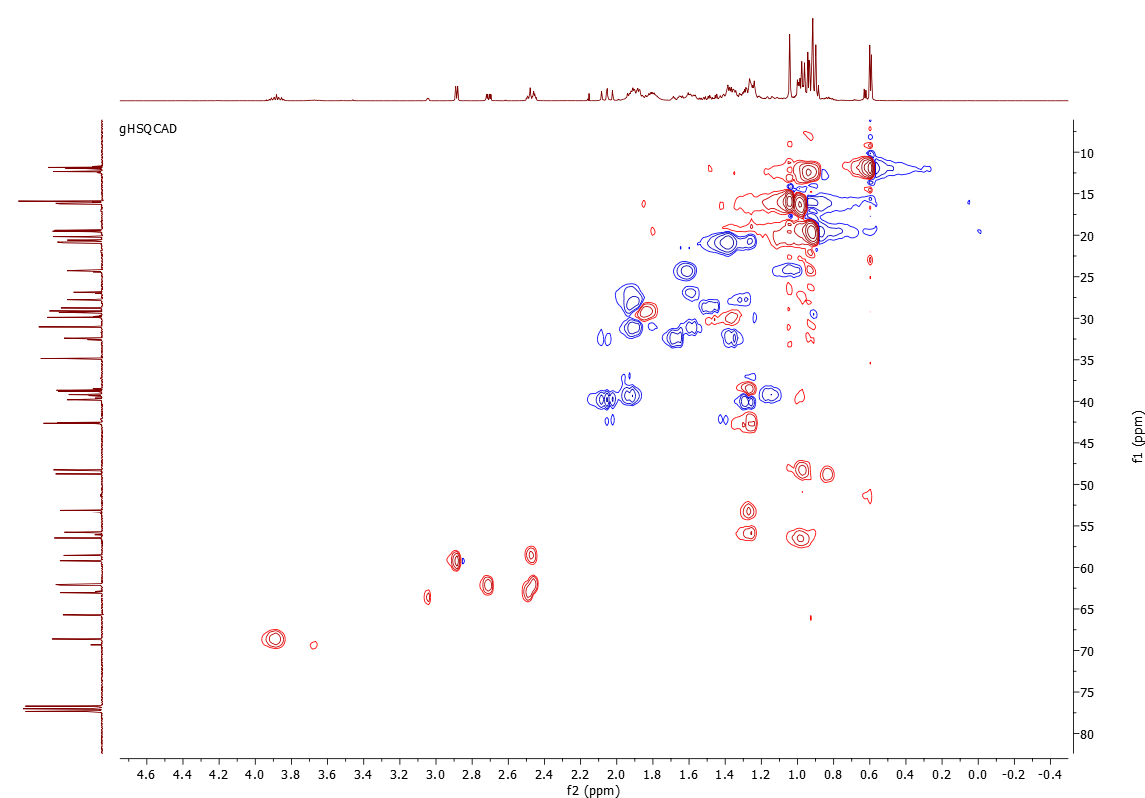
**

**Appendix 31:** HMBC spectrum of 5,6-Epoxystigmasta-3β,22,23-triol (**5**)

**
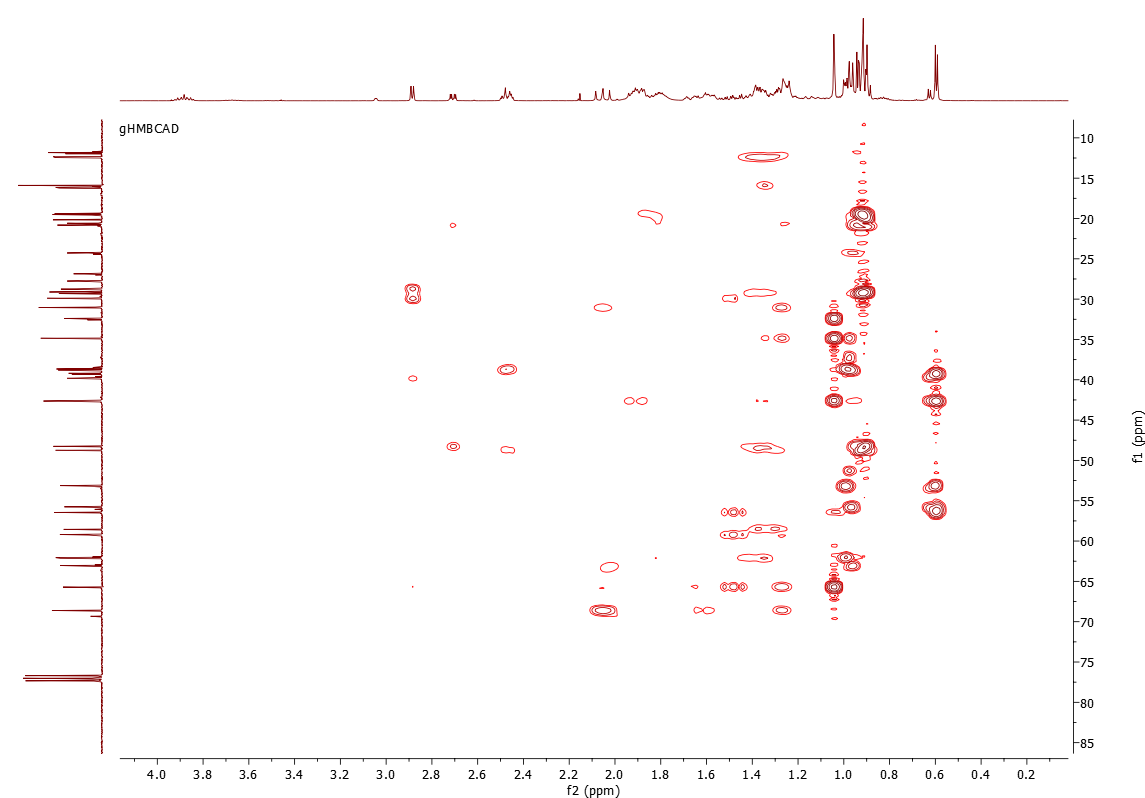
**

**Appendix 32:** IR spectrum of Stigmastane pentaol (**6**)

**
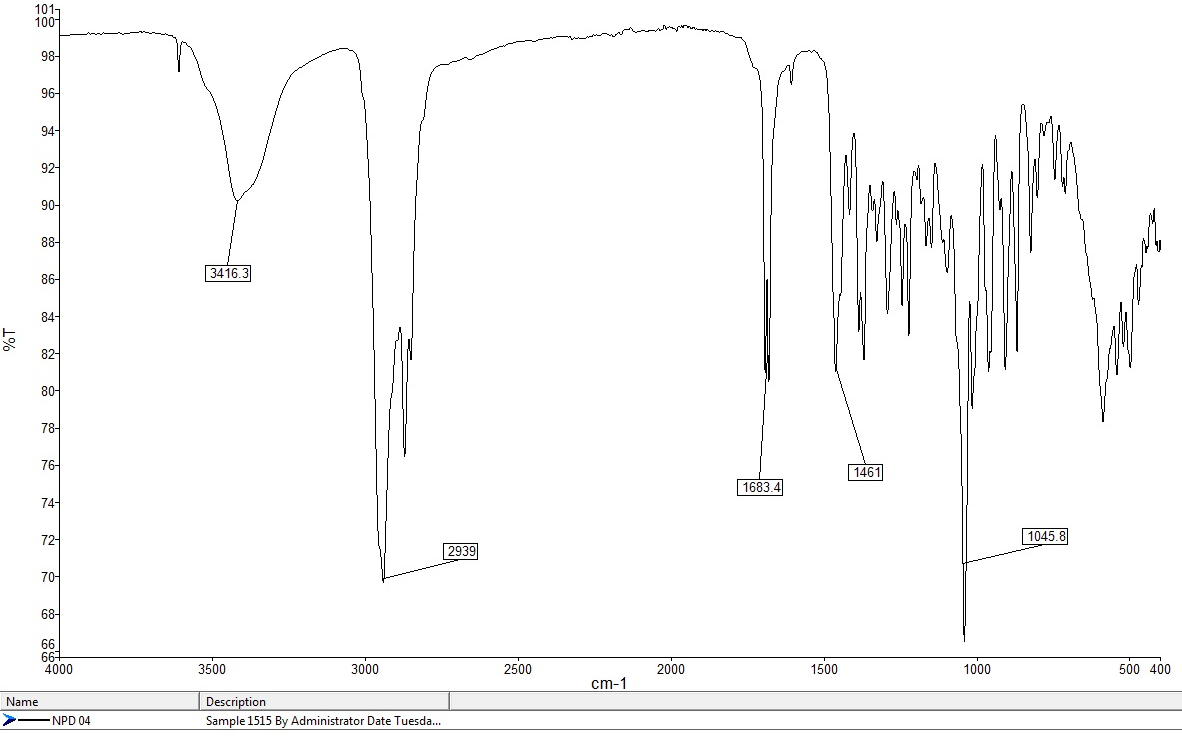
**

**Appendix 33:** MS spectrum of Stigmastane pentaol (**6**)

**
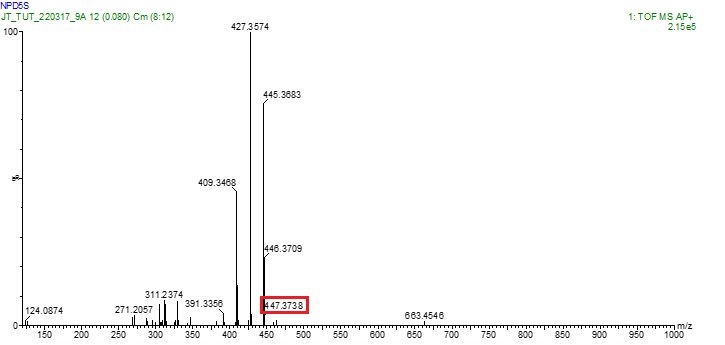
**

**Appendix 34:** ^1^H NMR spectrum of Stigmastane pentaol (**6**) in CDCl_3_

**
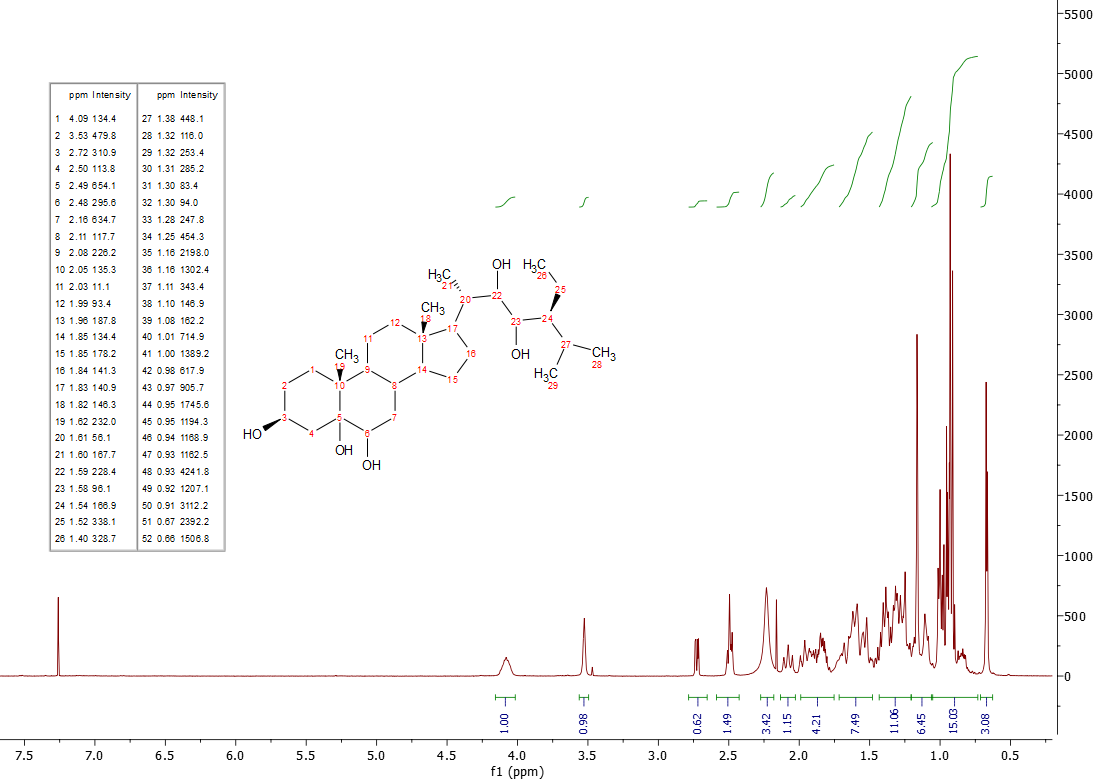
**

**Appendix 35:** ^13^C NMR spectrum of Stigmastane pentaol (**6**) in CDCl_3_

**
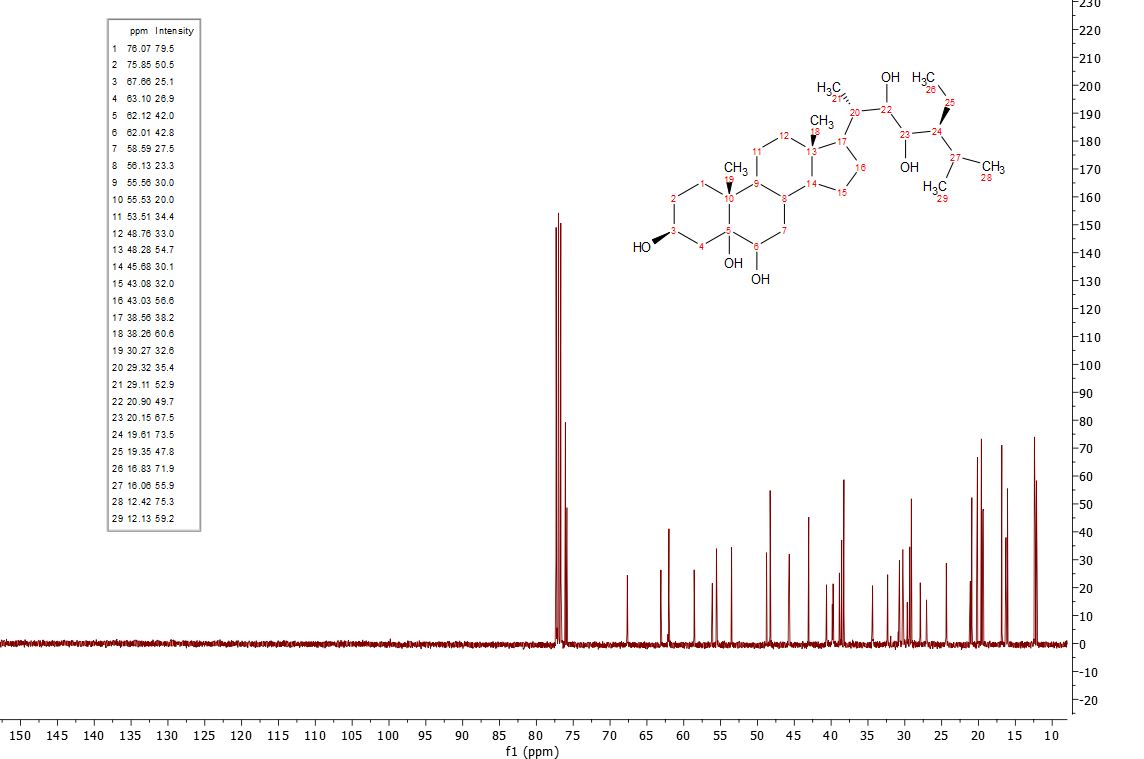
**

**Appendix 36:** HSQC spectrum of Stigmastane pentaol (**6**)

**
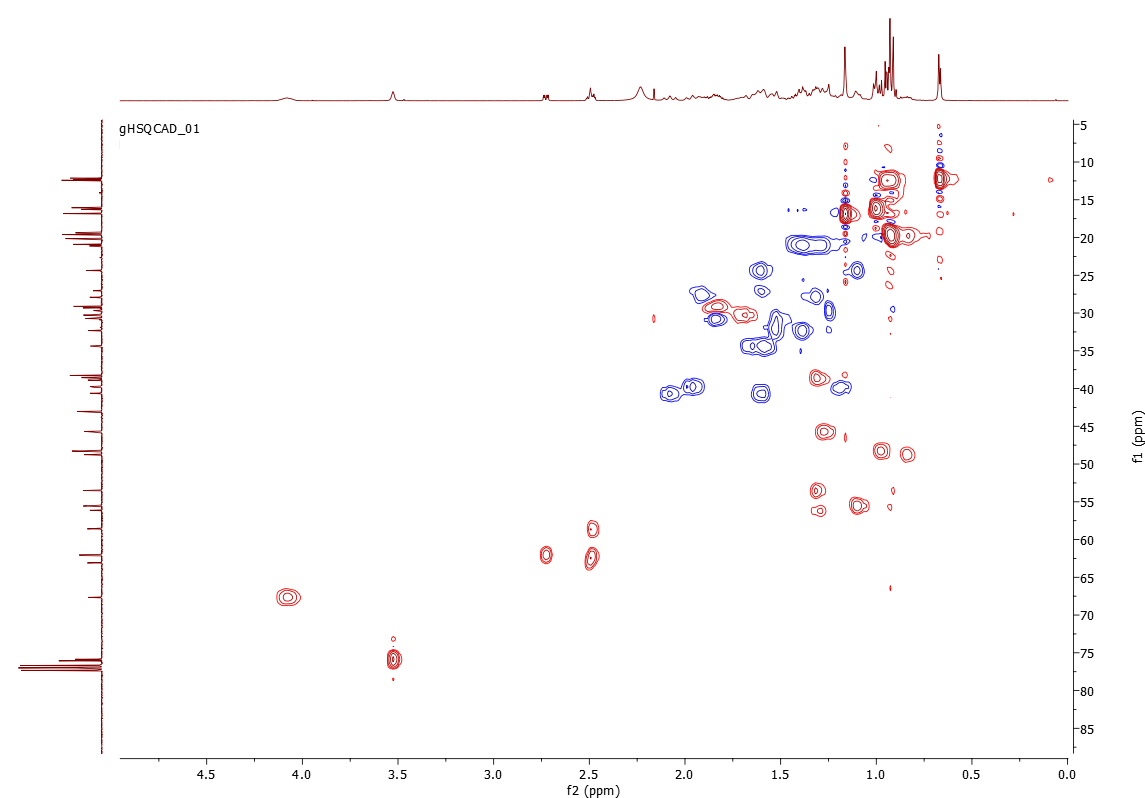
**

**Appendix 37:** HMBC spectrum of Stigmastane pentaol (**6**)

**
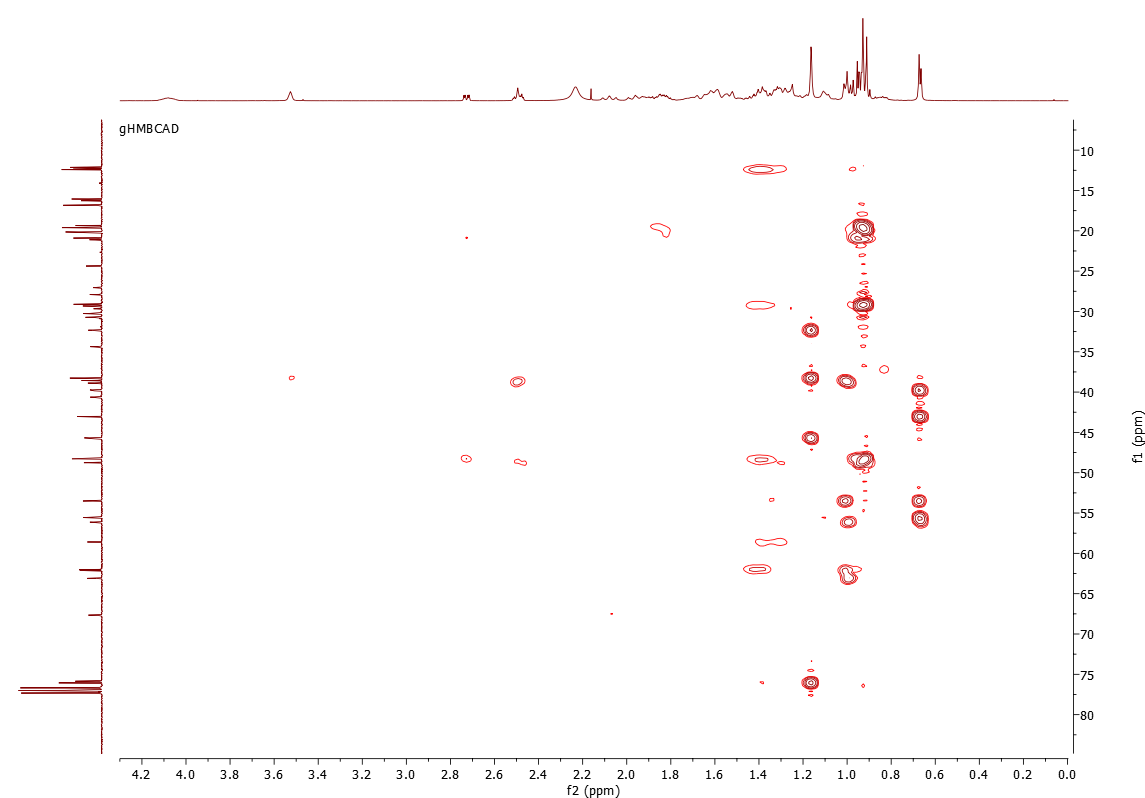
**

**Appendix 38:** IR spectrum of Stigmasta-5-en-3,7-dion-22,23-diol (**7**)

**
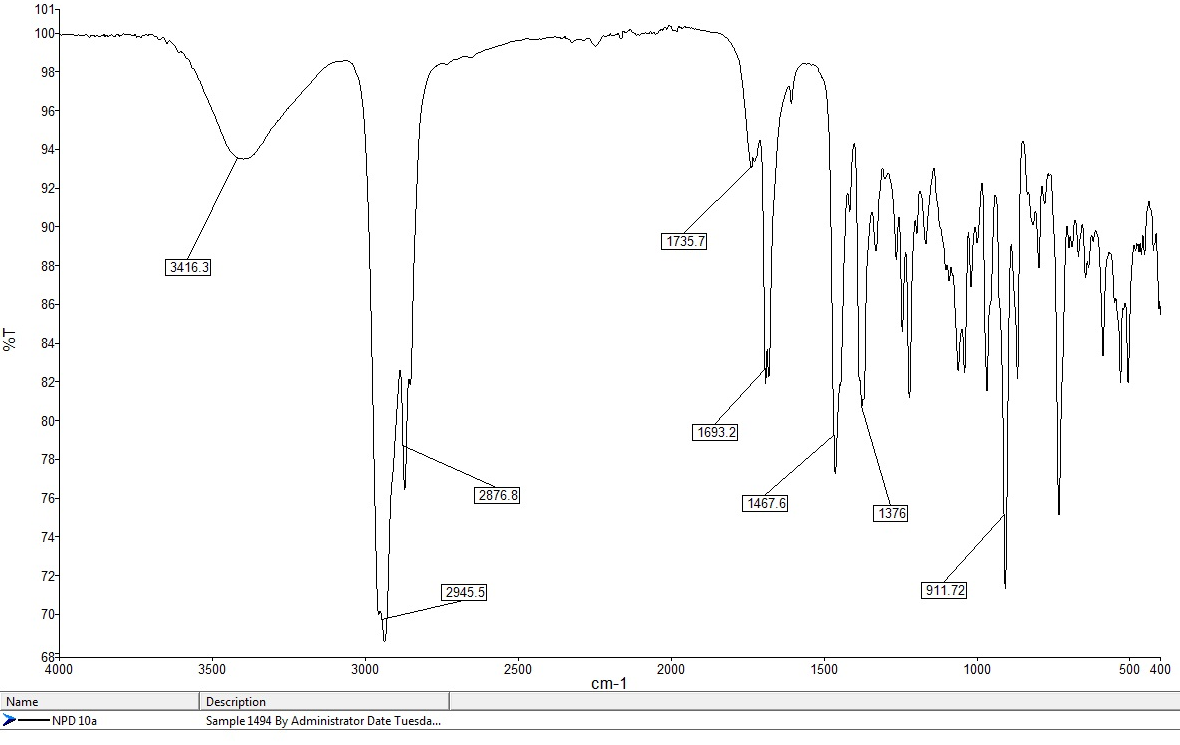
**

**Appendix 39:** MS spectrum of Stigmasta-5-en-3,7-dion-22,23-diol (**7**)

**
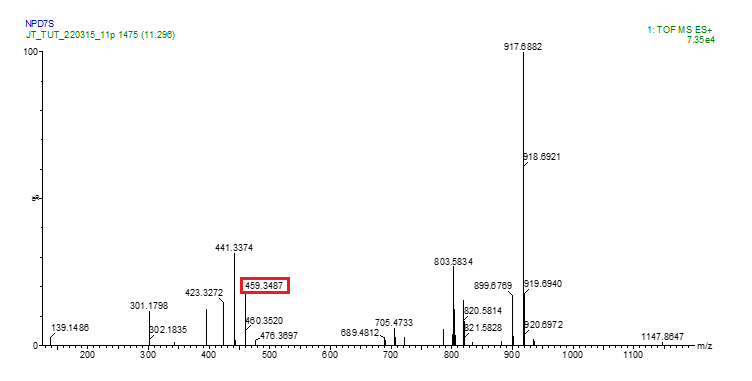
**

**Appendix 40:** ^1^H NMR spectrum of Stigmasta-5-en-3,7-dion-22,23-diol (**7**) in CDCl_3_

**
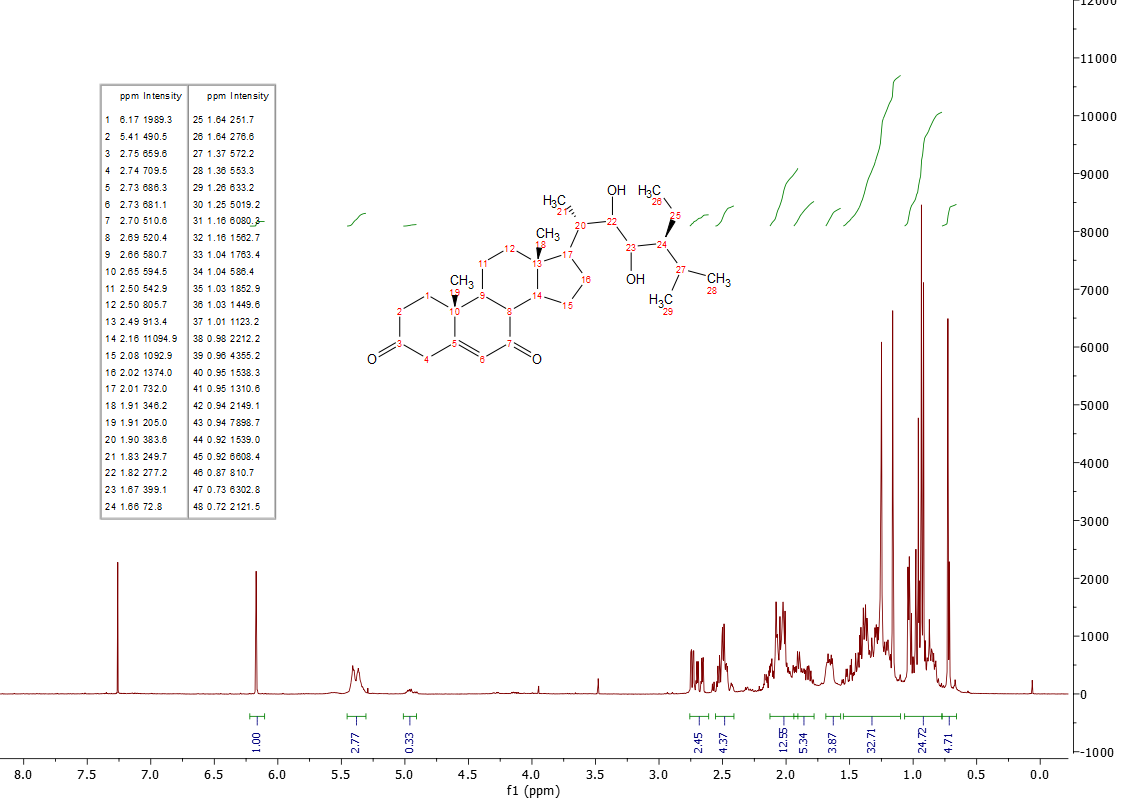
**

**Appendix 41:** ^13^C NMR spectrum of Stigmasta-5-en-3,7-dion-22,23-diol (**7**) in CDCl_3_

**
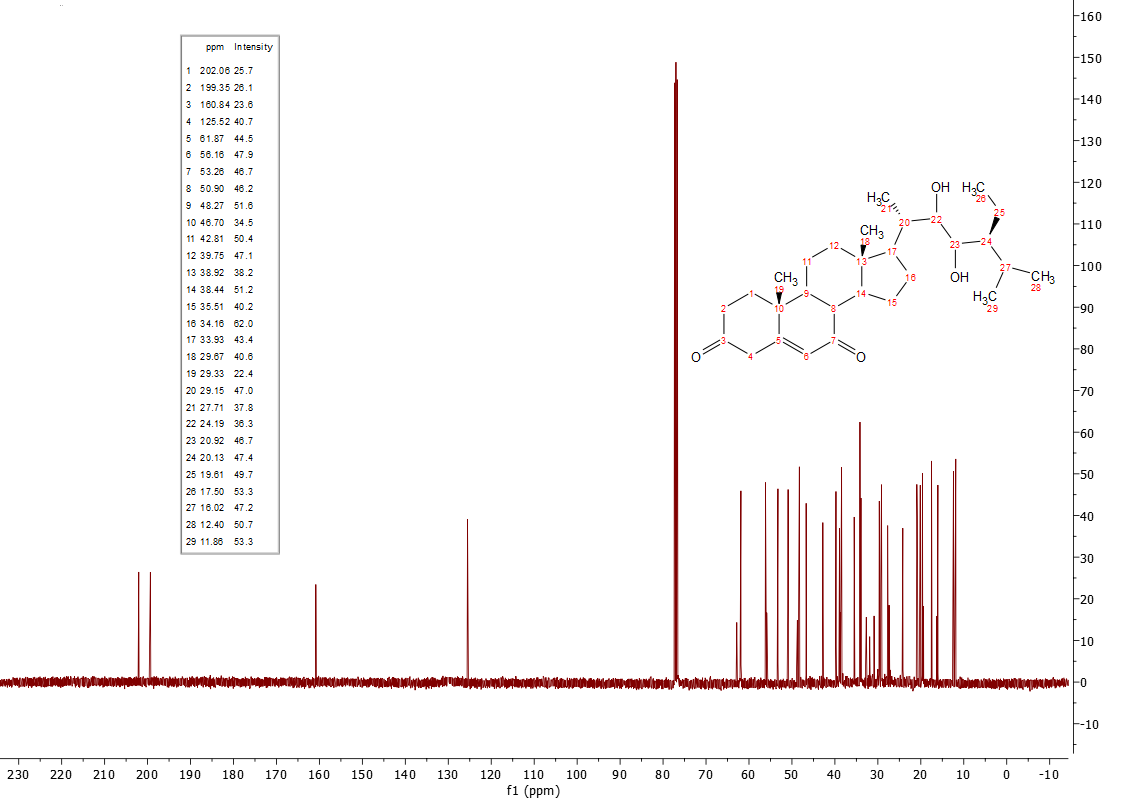
**

**Appendix 42:** DEPT spectrum of Stigmasta-5-en-3,7-dion-22,23-diol (**7**)

**
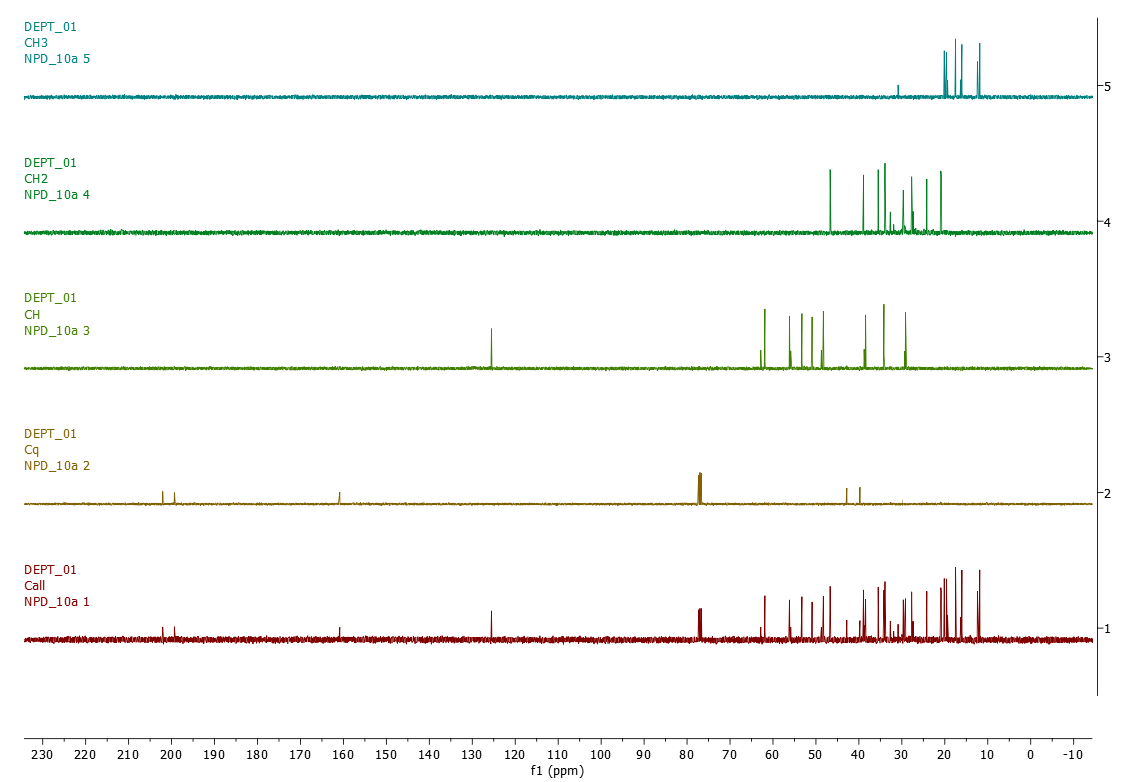
**

**Appendix 43:** HSQC spectrum of Stigmasta-5-en-3,7-dion-22,23-diol (**7**)

**
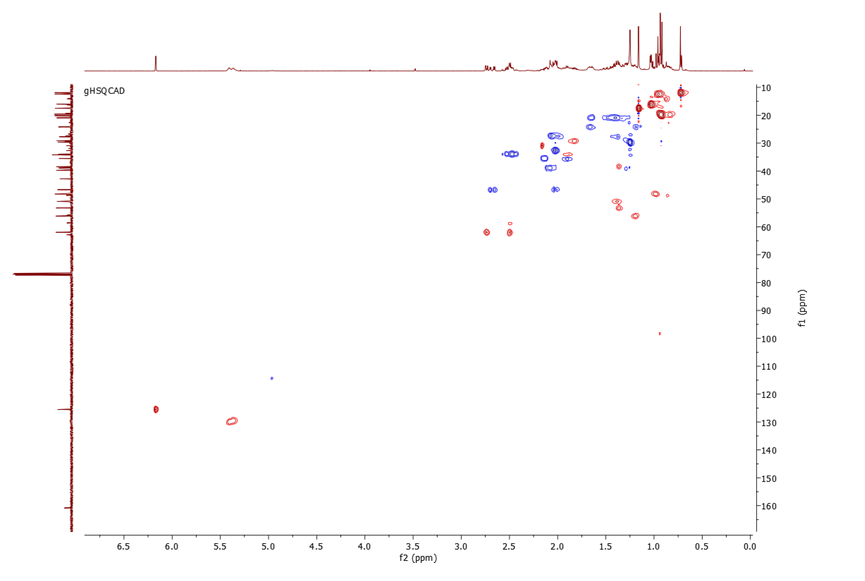
**

**Appendix 44:** HMBC spectrum of Stigmasta-5-en-3,7-dion-22,23-diol (**7**)

**
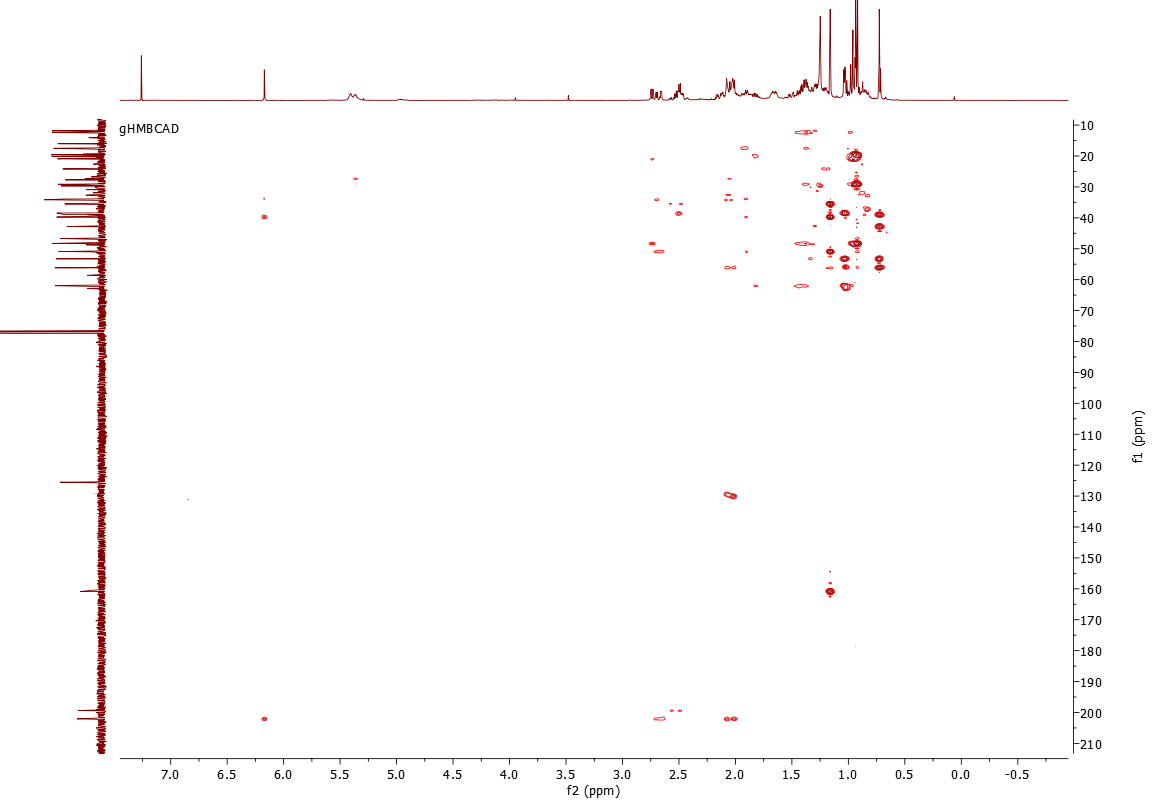
**

**Appendix 45:** IR spectrum of Stigmasta-3,7-dion-5,6,22,23-ol (**8**)

**
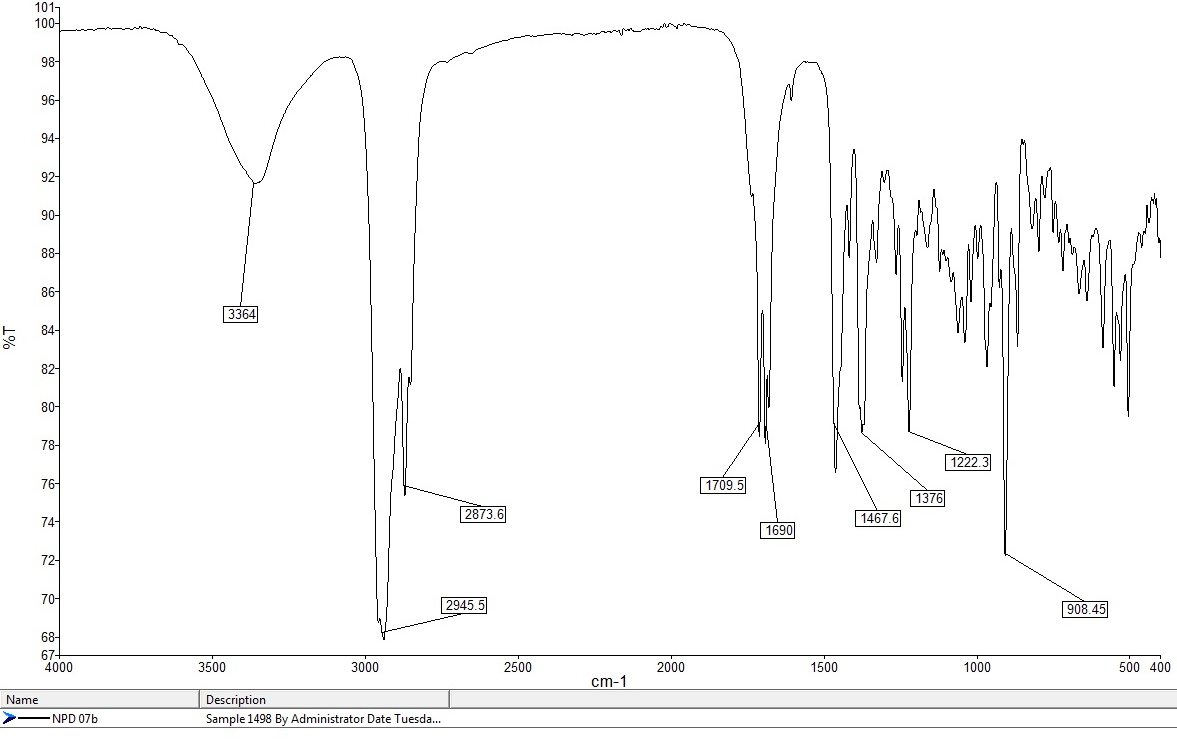
**

**Appendix 46:** MS spectrum of Stigmasta-3,7-dion-5,6,22,23-ol (**8**)

**
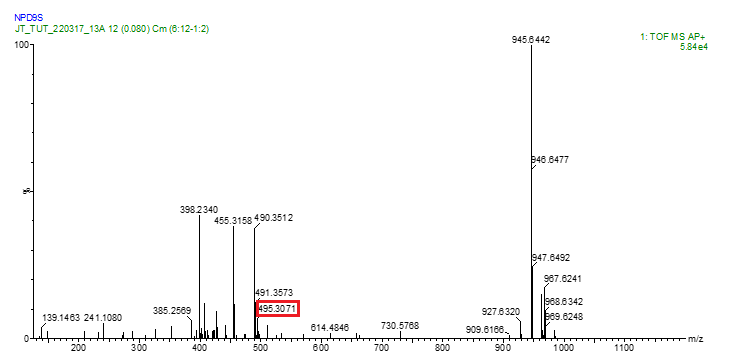
**

**Appendix 47:** ^1^H NMR spectrum of Stigmasta-3,7-dion-5,6,22,23-ol (**8**) in CDCl_3_

**
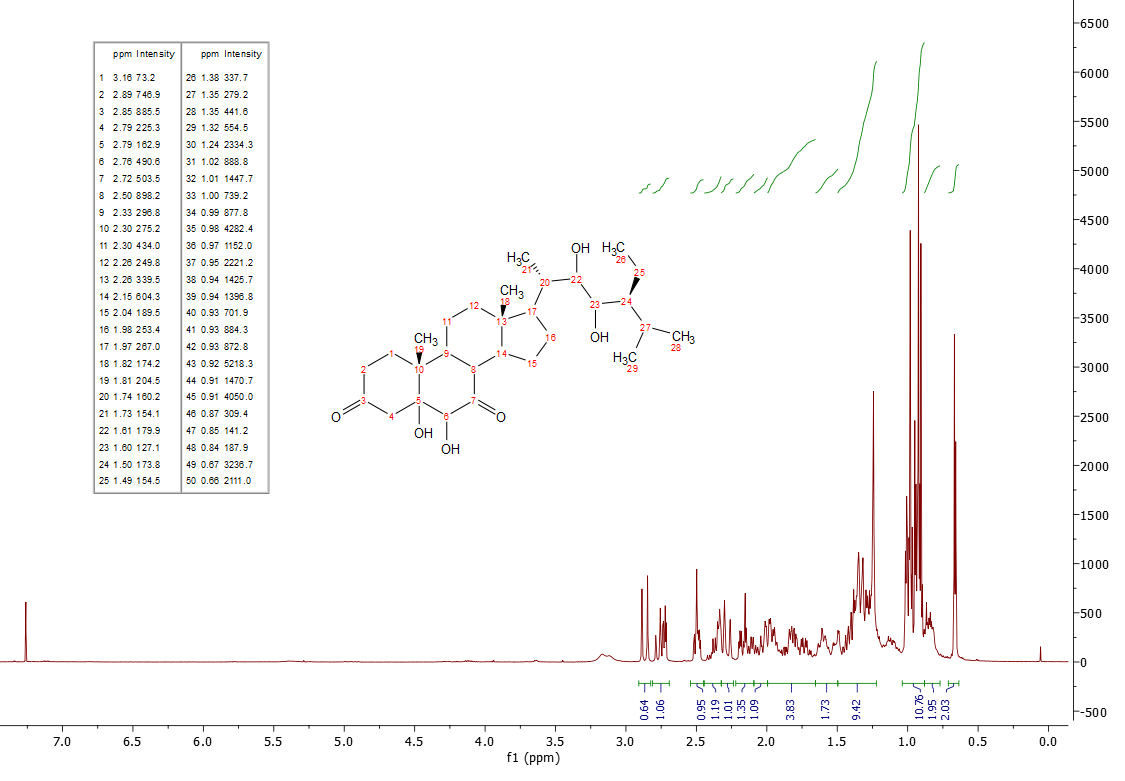
**

**Appendix 48:** ^13^C NMR spectrum of Stigmasta-3,7-dion-5,6,22,23-ol (**8**) in CDCl_3_

**
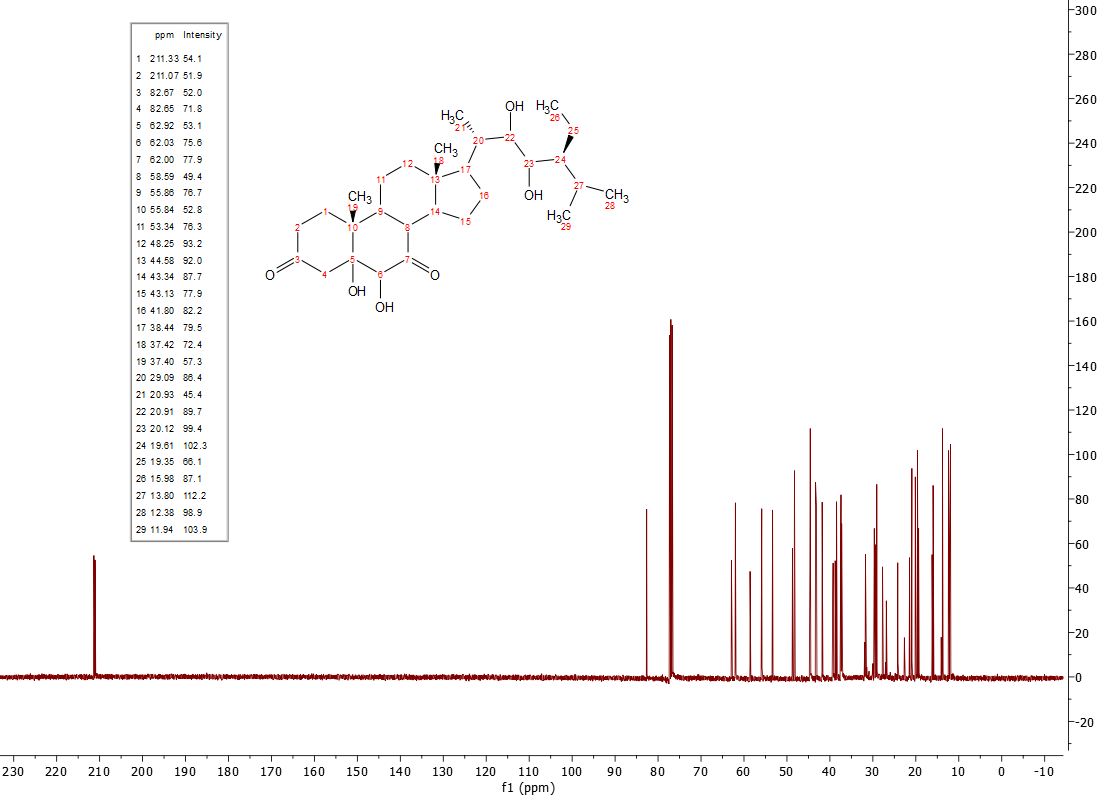
**

**Appendix 49:** HSQC spectrum of Stigmasta-3,7-dion-5,6,22,23-ol (**8**)

**
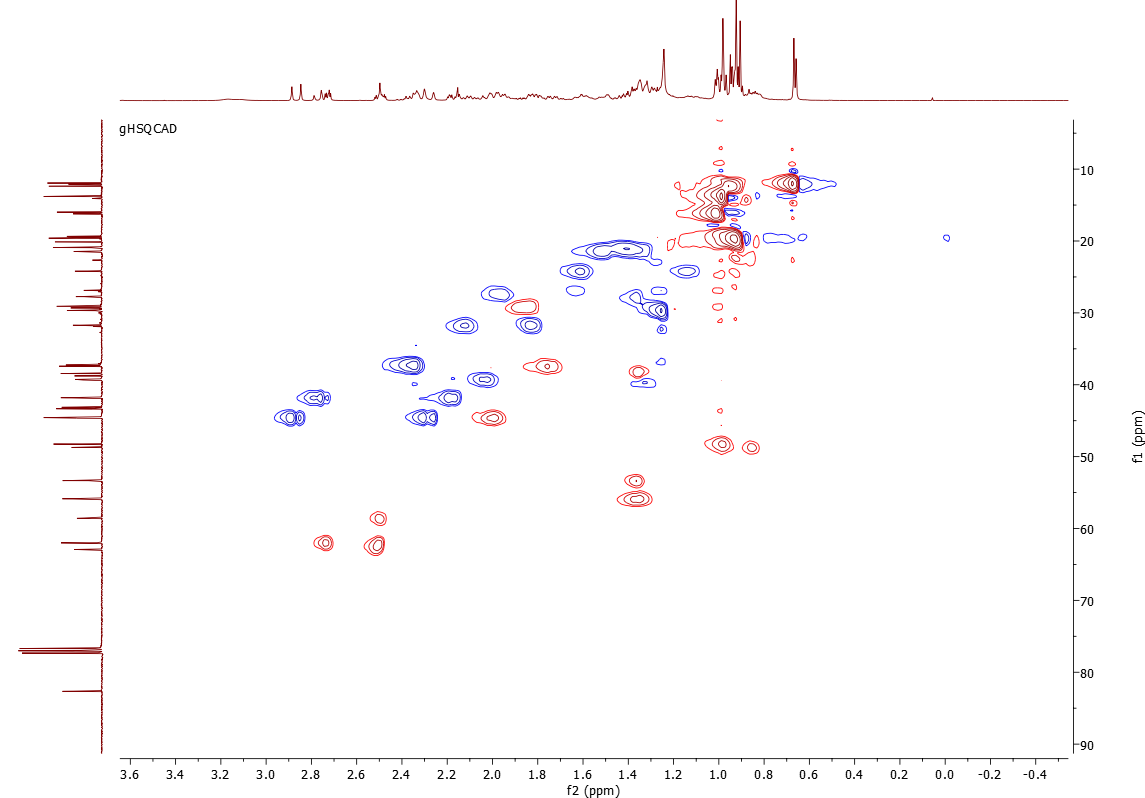
**

**Appendix 50:** HMBC spectrum of Stigmasta-3,7-dion-5,6,22,23-ol (**8**)

**
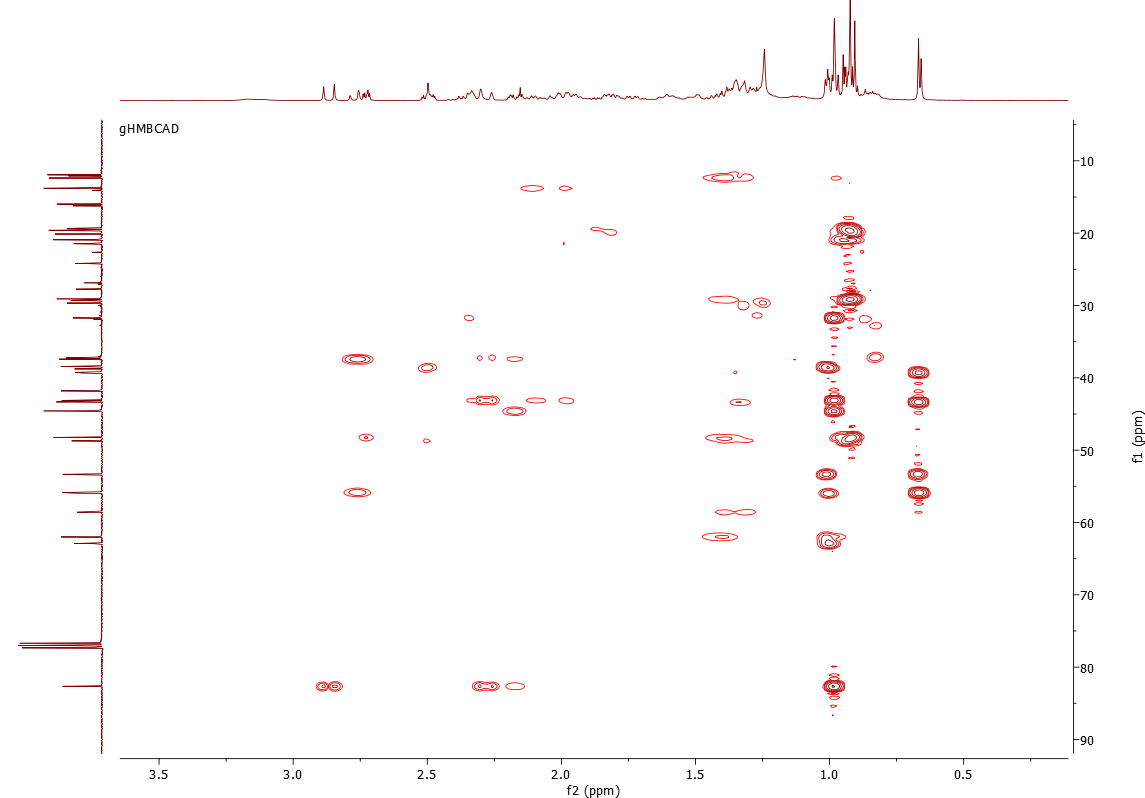
**

**Appendix 51:** IR spectrum of Stigmast-5-ene-3*β*,22,23-triol (**9**)

**
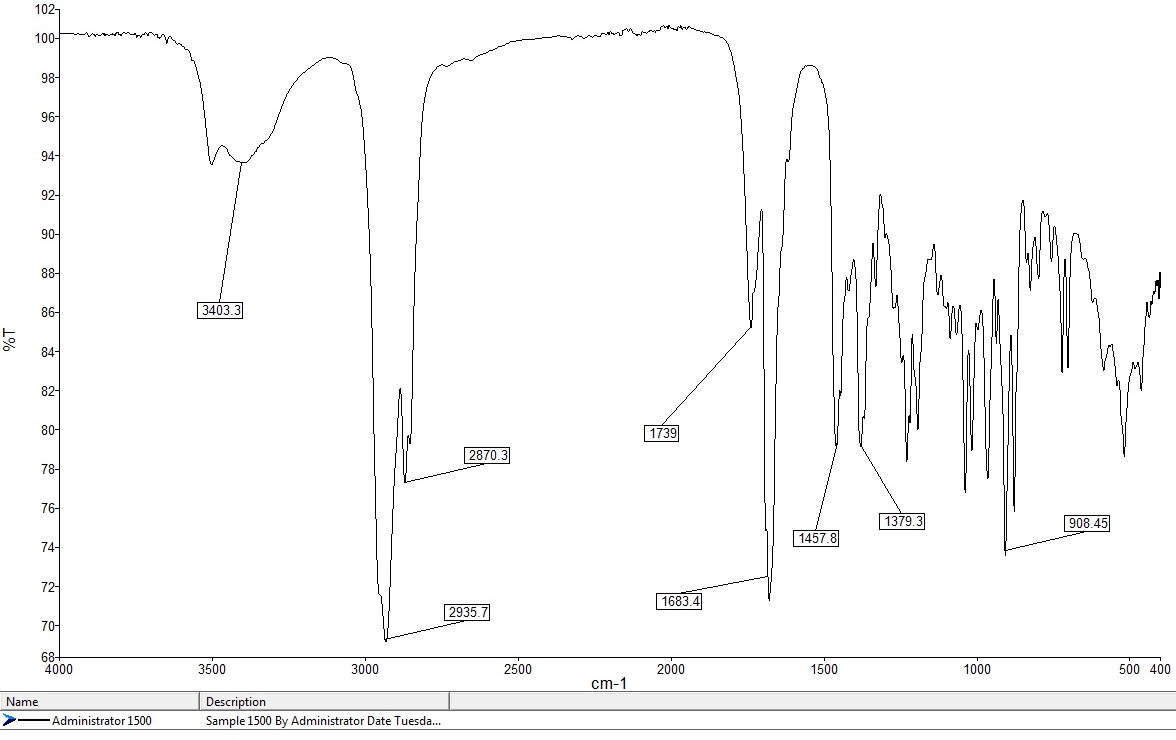
**

**Appendix 52:** MS spectrum of Stigmast-5-ene-3*β*,22,23-triol (**9**)

**
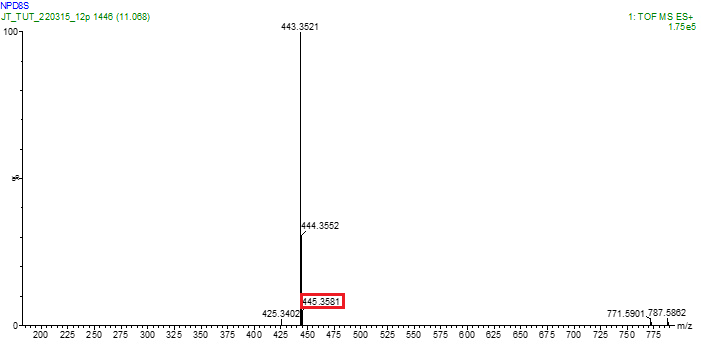
**

**Appendix 53:** ^1^H NMR spectrum of Stigmast-5-ene-3*β*,22,23-triol (**9**) in CDCl_3_

**
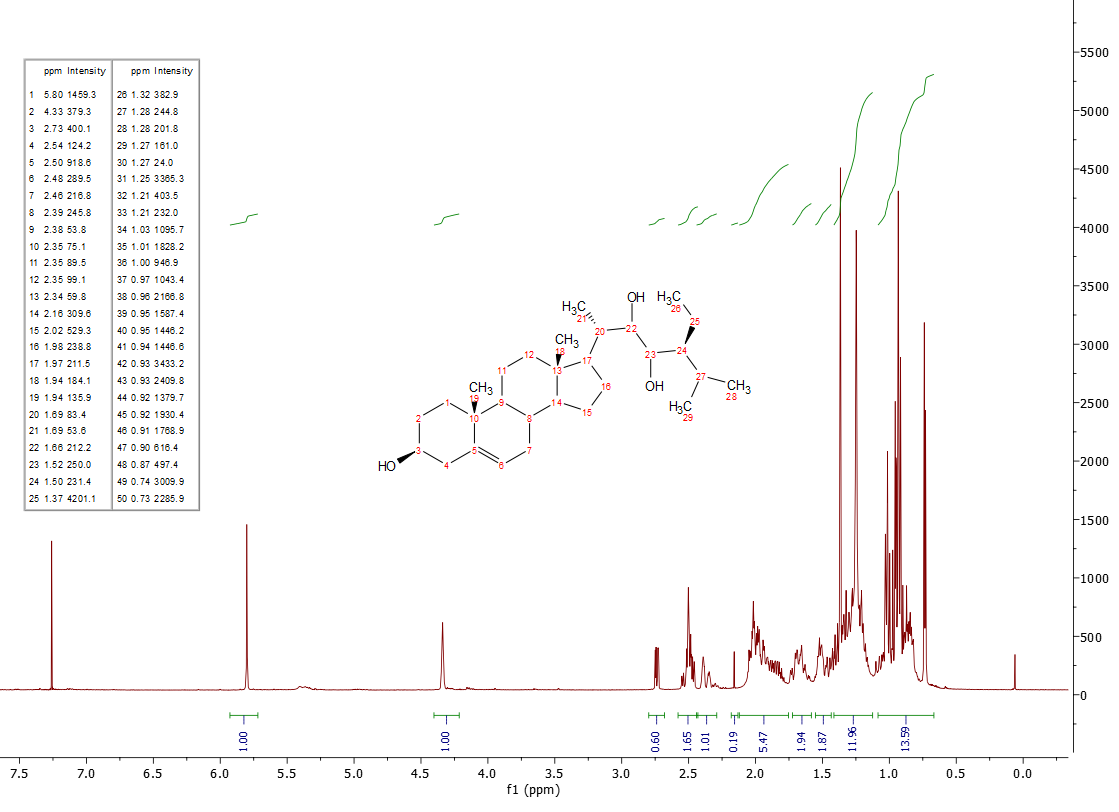
**

**Appendix 54:** ^13^C NMR spectrum of Stigmast-5-ene-3*β*,22,23-triol (**9**) in CDCl_3_

**
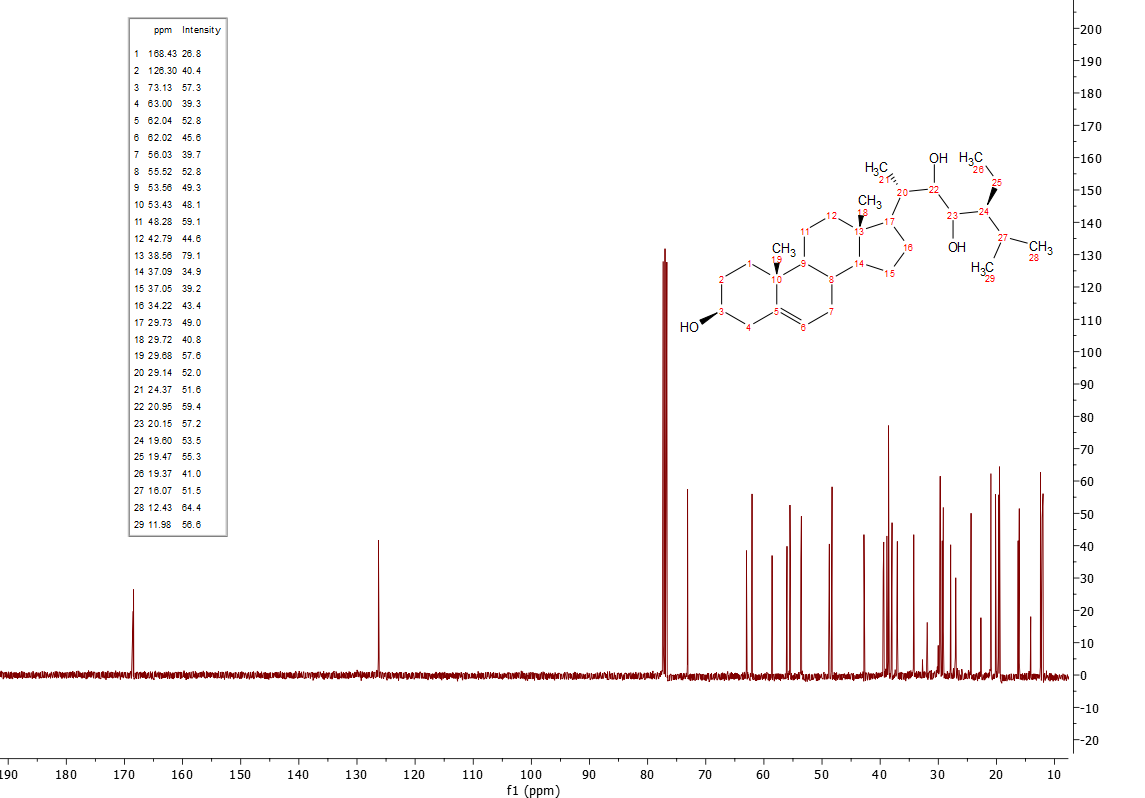
**

**Appendix 55:** DEPT spectrum of Stigmast-5-ene-3*β*,22,23-triol (**9**)

**
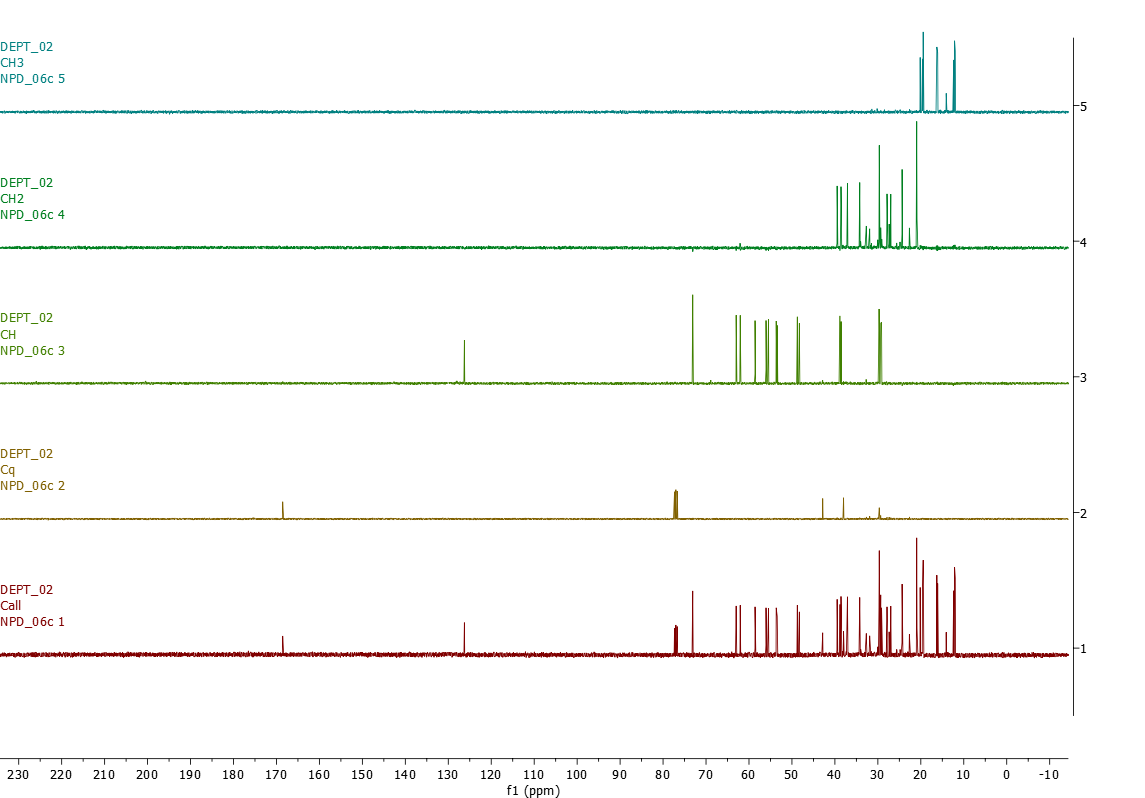
**

**Appendix 56:** HSQC spectrum of Stigmast-5-ene-3*β*,22,23-triol (**9**)

**
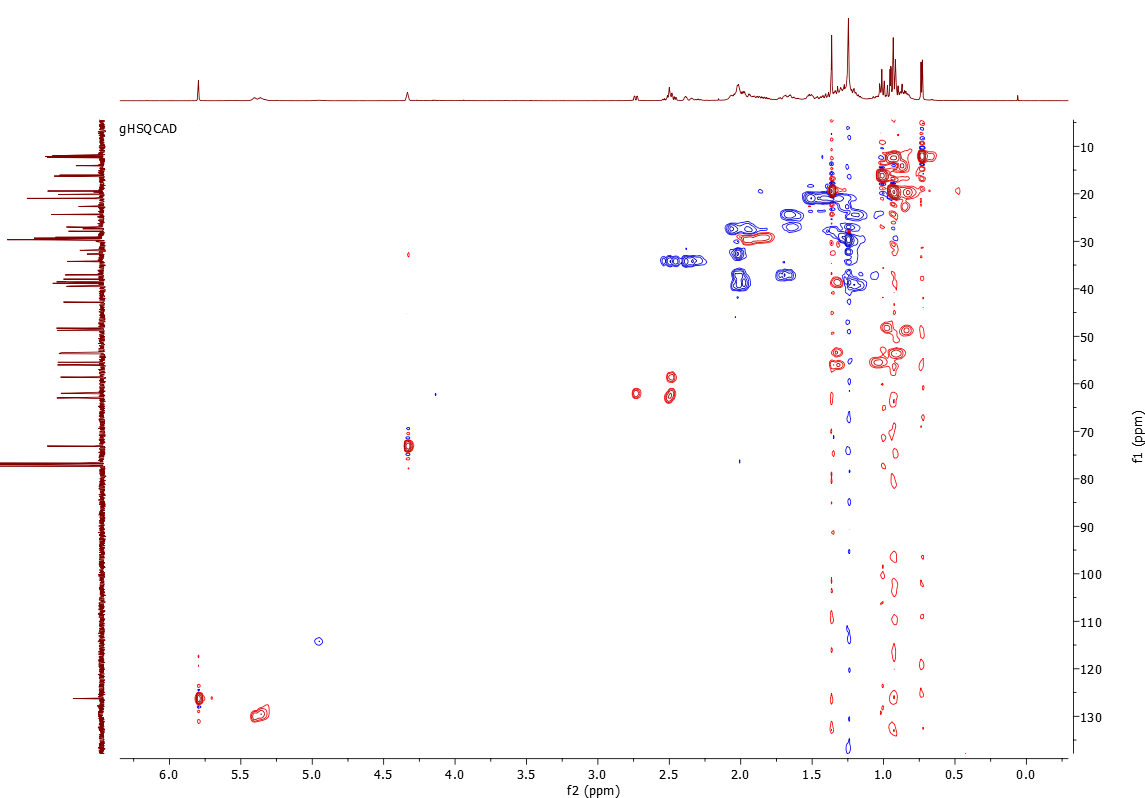
**

**Appendix 57:** HMBC spectrum of Stigmast-5-ene-3*β*,22,23-triol (**9**)

**
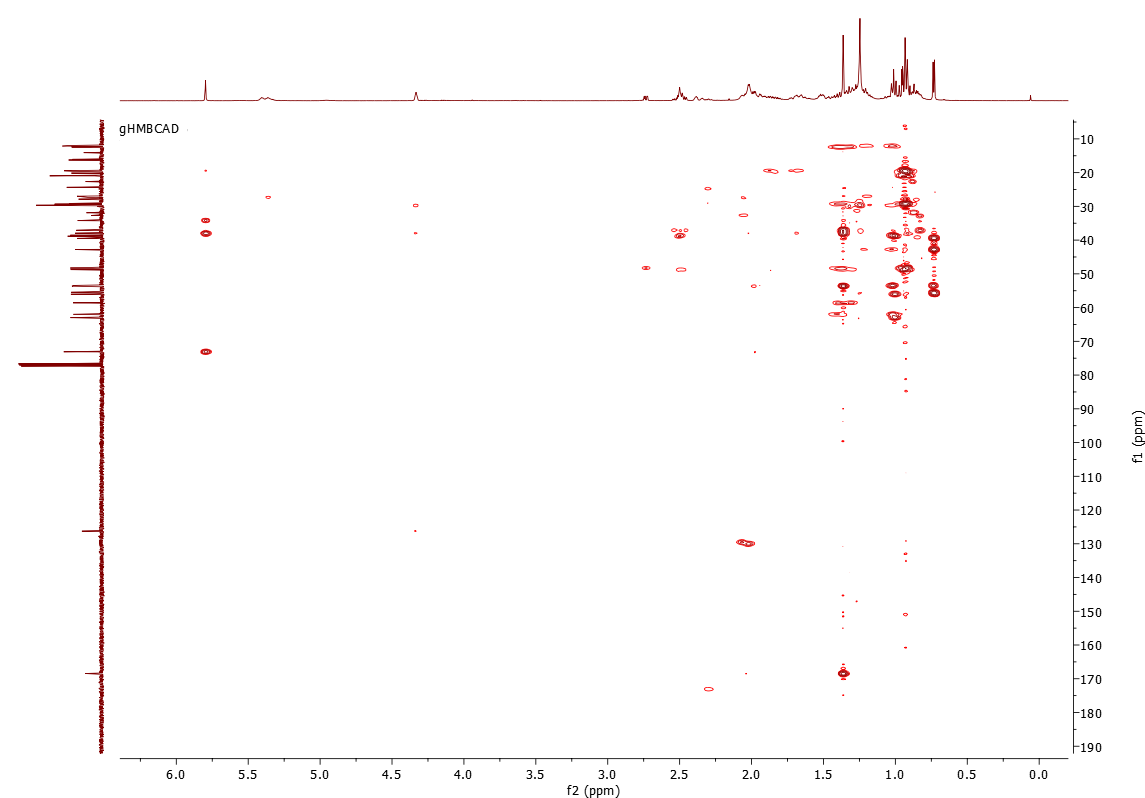
**
